# A scoping review of the barriers and facilitators to accessing and utilising mental health services across regional, rural, and remote Australia

**DOI:** 10.1186/s12913-023-10034-4

**Published:** 2023-10-04

**Authors:** Bianca E. Kavanagh, Kayla B. Corney, Hannah Beks, Lana J. Williams, Shae E. Quirk, Vincent L. Versace

**Affiliations:** 1 Deakin Rural Health, School of Medicine, Deakin University, Princes Highway, Warrnambool, VIC 3280 Australia; 2 Institute for Mental and Physical Health and Clinical Translation, School of Medicine, Deakin University, Barwon Health, Geelong, VIC Australia; 3 Institute of Clinical Medicine, Psychiatry, University of Eastern Finland, Kuopio, Finland; 4 Institute of Clinical Medicine, Kuopio Musculoskeletal Research Unit (KMRU), University of Eastern Finland, Kuopio, Finland

**Keywords:** Mental health, Rural health, Australia, Mental health services, Rural Health Services

## Abstract

**Background:**

Inadequate healthcare access and utilisation are implicated in the mental health burden experienced by those living in regional, rural, and remote Australia. Facilitators that better enable access and utilisation are also reported in the literature. To date, a synthesis on both the barriers and facilitators to accessing and utilising mental health services within the rural Australian context has not been undertaken. This scoping review aims to (1) synthesise the barriers and facilitators to accessing and utilising mental health services in regional, rural, and remote Australia, as identified using the Modified Monash Model; and (2) better understand the relationship between barriers and facilitators and their geographical context.

**Methods:**

A systematic search of Medline Complete, EMBASE, PsycINFO, Scopus, and CINAHL was undertaken to identify peer-reviewed literature. Grey literature was collated from relevant websites. Study characteristics, including barriers and facilitators, and location were extracted. A descriptive synthesis of results was conducted.

**Results:**

Fifty-three articles were included in this scoping review. Prominent barriers to access and utilisation included: limited resources; system complexity and navigation; attitudinal and social matters; technological limitations; distance to services; insufficient culturally-sensitive practice; and lack of awareness. Facilitators included person-centred and collaborative care; technological facilitation; environment and ease of access; community supports; mental health literacy and culturally-sensitive practice. The variability of the included studies precluded the geographical analysis from being completed.

**Conclusion:**

Both healthcare providers and service users considered a number of barriers and facilitators to mental health service access and utilisation in the regional, rural, and remote Australian context. Barriers and facilitators should be considered when re-designing services, particularly in light of the findings and recommendations from the Royal Commission into Victoria’s Mental Health System, which may be relevant to other areas of Australia. Additional research generated from rural Australia is needed to better understand the geographical context in which specific barriers and facilitators occur.

**Supplementary Information:**

The online version contains supplementary material available at 10.1186/s12913-023-10034-4.

## Introduction

The mental health of Australians who live in regional, rural, and remote Australia is an ongoing concern [1]. Poor healthcare access is one of the key determinants of adverse mental health outcomes, with access issues being more pronounced in regional, rural, and remote Australia (hereafter referred to as *rural*, in line with the Australian Government’s definition under the Rural Health Multidisciplinary Training [RHMT] Program [2]), compared to metropolitan Australia [3]. People living in rural Australia often face difficulties in obtaining healthcare, and this care is often delayed and more expensive for the patient [4]. These difficulties in accessing and utilising healthcare are implicated in the higher mental disorder burden experienced by those living in rural Australia, shown by the higher rates of suicide, compared with major cities [5]. Moreover, this group is less likely than those living in major cities to take-up and complete mental health treatment [6]. Workforce maldistribution plays a role in these health inequalities [7–10], with more clinical full time equivalent (FTE) mental health professionals working in major cities, compared with rural areas (i.e., 92 vs. 30–80 mental health nurses, 15 vs. 2–6 psychiatrists, and 90 vs. 15–55 psychologists per 100,000/population) [3]. Other areas of the health workforce are similarly maldistributed across the country (i.e., 403 vs. 223–309 clinical FTE medical practitioners and 531 vs. 382–469 clinical FTE allied health professionals per 100,000/population in major cities versus rural areas) [11].

There are a number of factors that are implicated — both directly and indirectly — in the access and utilisation of mental health services, and these factors may be pertinent to the level of remoteness experienced. This includes particular aspatial (i.e., social) and spatial (i.e., geographical) dimensions [12, 13]. Aspatial dimensions consist of the factors that affect the *affordability*, *acceptability*, *accommodation*, and *awareness* of healthcare access. In the rural context of Australia, this tends to relate to social matters [14, 15] including stoicism, low help-seeking behaviours, and confidentiality concerns [16]. Spatial dimensions are concerned with the *availability* and *accessibility* of service access, including geographical isolation [14], service delivery capacity [17] [18], and dual-roles [14] (i.e., the intersection of professional and personal relationships) in rural areas. While here we define access as factors that pertain to the attributes/expectations of the individual and their alignment with the provider/services [12], other models conceptualise access as the opportunity to identify healthcare needs, seek services, reach resources, obtain or use services, and have the need for services fulfilled [19]. Utilisation refers to the generation of a healthcare plan throughout a healthcare encounter, as well as its implementation and follow-through [20].

Conceivably, mitigating the barriers and augmenting the facilitators to the utilisation of mental health services may be particularly important when considering the obstacles that people from rural areas face when accessing services. One previous study on rurally-based Australian adolescents suggested that barriers to accessing services, such as social exclusion and ostracism by members of their community, also likely prevented the continued utilisation of services and negatively affected treatment outcomes [21]. Cheesmond et al. [22], in a review of residents in rural Australia, Canada, and the United States of America, highlighted a link between sociocultural rurality, rural identity, and help-seeking behaviour. Cheesmond et al. [22] suggested that specific place-sensitive approaches are needed to overcome barriers to help-seeking, and that a greater understanding of help-seeking in the rural context is required. This includes further exploration of rurality as a concept, conducting research within diverse environments, allowing participants to contextualise barriers to help-seeking, and exploring the co-existence of multiple help-seeking barriers. Parallel to this, a paucity of research has focussed on the facilitators to accessing and utilising mental health services in rural Australia.

To the authors’ knowledge, no previous reviews have specifically focussed on understanding the barriers and facilitators to accessing and utilising mental health services within the rural Australian context. A scoping review was chosen as the preferred approach to this work because of the emerging and cross-disciplined nature of the research. The aim of this scoping review is to: (1) explore the barriers and facilitators to accessing and utilising mental health services for Australians living in rural areas; and (2) better understand the relationship between barriers and facilitators and their geographical context.

## Method

This scoping review conforms to the guidelines put forward by Arksey and O’Malley [23], follows the Preferred Reporting Items for Systematic Reviews and Meta-Analysis extension for scoping reviews (PRISMA-ScR) [24], and a published protocol [25].

### Eligibility criteria

The scope of this review was intentionally broad to allow explanation of the nature and extent of the literature describing the barriers and facilitators to accessing and utilising mental health services across regional, rural, and remote Australia. Articles were eligible for inclusion if they met the following criteria:

- Included individuals with a diagnosed mental disorder, experienced mental health issues, or were part of a mental health community service; or included healthcare providers that provided diagnostic, assessment, or treatment services for mental health issues.
- Explained obstacles that impeded the uptake, quality, or level of mental health services being accessed or described facilitators that allowed the uptake, quality, or level of mental health services being received.
- Included service users, healthcare providers, or services that were based in regional, rural, or remote Australia according to the Modified Monash Model (MMM) 2–7 (*regional centres* to *very remote communities*) [4] (i.e. the current RHMT definition of rural).

The population/concept/context (PCC) framework was used to generate the eligibility criteria for this scoping review and is described in Table 1. The eligibility criteria for this review varied slightly from the published protocol [25]. In this review, we included pharmacists as healthcare providers, as it was identified that pharmacists play a key role in mental health services in some rural areas. We excluded mental health programs and health promotion activities that were considered to be a “structured activity” delivered by a service, reviews, viewpoints, declarations, tailpieces, frameworks, and commentaries. We also excluded articles that did not provide sufficient detail to describe the barriers or facilitators to accessing or utilising services, as well as articles that pooled results across participants from metropolitan and regional/rural/remote areas. The only exception to this was when authors referred to the study setting as regional/rural/remote, but upon further investigation using the health workforce locator [26] (see Sect. 2.8*Geographical analysis*), the location was deemed to be metropolitan according to the MMM [4] — this exception was allowed due to the differences in geographical models applied to Australian health research [27, 28]. Separately, we decided to include articles that reported on the barriers and/or facilitators of a specific rural mental health service implementation activity or service model, as we felt that these articles offered important insights that may be translated to new service initiatives or research activities.

**Table 1:** Population/Concept/Context

	Inclusion	Example	Exclusion
P	Patients/individuals with mental health issues/concerns of any age	Diagnosed mental disorders: • Schizophrenia (spectrum) and other psychotic disorders • Depressive disorders • Bipolar and related disorders • Anxiety disorders • Obsessive-compulsive and related disorders • Trauma- and stressor-related disorders • Somatic and related disorders • Dissociative disorders • Feeding and eating disorders • Disruptive, impulse control, and conduct disorders • Substance-related and additive disorders • Personality disorders Mental health issues: • Psychological distress indicated via validated measure • “At-risk” groups (e.g., where mental health services have been sought/warranted but a diagnosis has not yet been made) • Mental disorders not otherwise specified Part of mental health/community service: • Adult mental health service • Child and adolescent mental health service • Community mental health organisation	Neurodevelopmental disorders Elimination disorders Sleep-wake disorders Sexual dysfunctions Gender dysphoria Neurocognitive disorders Paraphilic disorders
	Healthcare providers providing diagnostic/assessment/treatment for mental health issues	• Medical specialists (e.g., general practitioners and psychiatrists) • Allied health professionals (e.g., psychologists, social workers, counsellors) • Nurses and nurse practitioners • Drug and alcohol workers • Community mental health workers (i.e., workers who provide social/housing/occupational support) • Peer-workers • Pharmacists	Healthcare providers who do not specifically diagnose/assess/treat individuals with mental health issues
C	Barriers	Obstacles that obstruct the uptake of mental health services or factors that prevent the quality/level of care being accessed • Confidentiality concerns • Fear of stigma • Poor mental health literacy • Geographic isolation • Limited appointment availability • High cost of service	Factors that are not considered to be barriers
	Facilitators	Factors that permit the uptake of mental health services or factors that allow the appropriate amount/quality of care to be received: • Telehealth availability • Free/low cost of service • Appointment timeliness • Safe and supportive environment • Culturally competent healthcare providers • Mentors to assist with system navigation	Factors that are not considered to be facilitators
	Access factors	Factors that measure of the alignment between healthcare provider/services and the characteristics/expectations of clients: • Aspatial dimensions (i.e., affordability, acceptability, and accommodation) • Spatial dimensions (i.e., availability and accessibility)	Factors that are not considered to be related to access
	Utilisation factors	Factors that affect the utilisation of healthcare services, including the implementation of subsequent healthcare encounters: • Effective information exchange • Satisfactory negotiation of a healthcare plan • Interpersonal relationship between the healthcare provider and the patient	Factors that are not considered to be related to the utilisation of mental health services
	Mental health services	• Services provided by hospitals (public and private) • Community-based services (i.e., Acute Community Intervention Service [ACIS], community care units (CCUs), Prevention and Recovery Centres (PARCs), and outpatient clinical treatment) • Mental Health Community Support Services (MHCSS) (e.g., services that are operated by non-government organisations) • Specialist mental health services (e.g., services provided specifically for individuals with certain mental health needs) • Outreach services • Day programs • Early intervention programs embedded within schools	All other health services, mental health programs, health promotion initiatives
C	Regional, rural, and remote areas of Australia	Areas classified as regional, rural, or remote Australia according to the MMM: • MM2 Regional Centres • MM3 Large Rural Towns • MM4 Medium Rural Towns • MM5 Small Rural Towns • MM6 Remote Communities • MM7 Very Remote Communities	Areas classified as a Major City in Australia according to the MMM: • MM1 Metropolitan Areas

### Information sources

The following databases were systematically searched: Medline Complete, EMBASE, PsycINFO, Scopus, and Cumulative Index of Nursing and Allied Health Literature (CINAHL). Websites of the Australian federal and state government’s Department of Health, Primary Health Network (PHN), key rural and remote peak bodies/agencies known to the authors from their collective experience on the topic, and Google were also searched to ascertain grey literature. The search was performed on 11th January 2022 and a 2012-current date filter was employed using the ‘start’ and ‘end’ publication year functions. Additional sources were identified through ‘snowball’ searching of included studies. Where needed, additional location information was obtained via a study’s first or corresponding author.

### Search

The search strategy was developed in consultation with two scholarly services librarians (JS and BK) to identify peer-reviewed studies and grey literature records. Relevant keywords, search terms, and wildcard symbols were applied to each database. An adapted search string was searched in Google using the advanced search function. The “all these words” and “any of these words” search options were engaged, and PDF files were requested. All (n = 11) pages of the search results were assessed for eligibility by one reviewer (BEK), and the research term agreed on their inclusion.

The full search strategy and grey literature sources are presented in Additional Table 1.

### Selection of sources of evidence

One reviewer (BEK) applied the search strategy to the databases and websites. Two reviewers (BEK and KBC) independently screened all articles using Covidence [29]. Where discrepancies concerning the eligibility of an article occurred, a meeting was held to determine consensus; if consensus could not be reached, a third reviewer (LJW) was consulted to make the final decision.

### Data charting process

To ensure that the data charting process was consistent with the research question, a charting form was developed and piloted by two authors (BEK and KBC). One author (BEK) then charted the data for each of the eligible articles, using Microsoft Excel.

### Data items

The following data items were extracted from eligible studies: author and year, study objective, study design, location, sample size, characteristics of participants, mental health diagnosis/issue and assessment method, healthcare provider type/role, barriers, facilitators, mental health service, regional/rural/remote area of Australia, and summary of findings (Additional Table 2). For literature that included participants from both metropolitan and regional/rural/remote areas, only information that pertained to those from regional/rural/remote areas was extracted, except for instances where statistical differences between groups were reported for comparison. Likewise, in instances where articles included participants who were eligible (e.g., healthcare providers) as well as participants who were ineligible (e.g., no evidence of mental health diagnosis/engagement with services), only information from eligible participants was extracted. First or corresponding authors of studies that did not specifically state where the study was conducted were contacted to provide additional location information.

### Synthesis of results

A descriptive synthesis was conducted by providing an overview of the included study characteristics, setting and target groups, and barries and facilitators. Links to aspatial and spatial access factors were also described, where relevant. The study characteristics are presented in Table 2 and the barriers and facilitators pertaining to each included study are presented in Additional Table 3. A quality appraisal of the included studies was not undertaken as scoping reviews aim to offer an overview or map of the pertinent evidence [30].

**Table 2:** Characteristics of the included studies

Author citation and location	Study setting	Study design	Study population	Mental health service context	
***Healthcare provider perspective***					
Barraclough et al. 2016; Lismore NSW	Lismore NSW (MM3)	Evaluation; mixed methods: documentary and quantitative evidence, qualitative interviews and meeting	N = 21; participants were nurse practitioner (NP) n = 1, senior health service managers n = 5, nursing leaders n = 2, manager of non-government organisation n = 1, mental health/drug and alcohol workers and community nurses/nurses from the emergency department (ED) n = 6, general practitioner (GP) n = 1, police superintendent n = 1, representative community-based organisation n = 1; sex: NR; majority ≥ 18 years (98%)	NP-led primary mental health service	
Beks et al. 2018; Warrnambool VIC	Portland (MM4), Hamilton (MM4), and Ararat (MM4) VIC	Qualitative interviews	N = 13; participants were registered nurses without postgraduate mental health qualifications; sex: female 100%; 25–34 years n = 1, 35–44 years n = 5, 45–54 years n = 4, ≥ 55 years n = 3	Acute mental health presentations in a rural emergency department and urgent care centres	
Clough et al. 2019; Southport QLD	Metropolitan, outer regional, rural Australia	Mixed methods: quantitative cross-sectional, qualitative interviews	Quantitative: n = 274, qualitative: n = 25; participants were medical doctors and stakeholders representing the Australian Medical Association, the Doctors’ Health Advisory Service, hospital-based medical education, and practice management; sex: female (73.4%), age M = 37.4, SD = 9.2 years	Perceptions of help-seeking for stress and burnout among medical doctors	
Cosgrave et al. 2015; Armidale NSW	Rural NSW	Qualitative interviews	N = 5; participants were community mental health mangers working in rural services; sex: NR; age NR	Community mental health services in rural Australia	
Cosgrave et al. 2018; Wangaratta VIC	Rural north-western NSW	Qualitative interviews	N = 25; participants were registered nurses n = 6, social workers n = 6 psychologists n = 4, occupational therapists (OT) n = 3, Aboriginal mental health workers n = 5, other workers n = 1; small town n = 9, medium town n = 3, large town n = 12, town NR n = 1; sex: NR; age NR	Community mental health services operated by NSW Health	
Crotty et al., 2012; Adelaide SA	Regional SA	Qualitative interviews	N = 10; participants were health and community service professionals working within local mental health and related services; sex: NR; age NR	Local mental health and related services	
De Silva et al. 2017; Lismore NSW	Northern Rivers Region NSW (MM5)	Qualitative interviews	N = 10; participants were GPs working in the Northern Rivers Region, NSW; sex: female 20%; age NR	Mental health services for mild to moderate depression in rural northern NSW	
Ellem et al. 2019; Brisbane QLD	Regional and rural NSW, regional QLD, regional and rural VIC	Qualitative interviews and focus groups	N = 43; participants were direct practitioners n = 25, supervisors n = 6, managers n = 9, or worked in policy/advocacy roles n = 3 within child and family welfare, Indigenous-specific, and mental health settings. Sex: NR, age NR	Services for youth with complex support needs, including family welfare, Indigenous-specific, and mental health services	
Evans et al. 2020; Port Macquarie NSW	Rural NSW	Qualitative focus groups	N = 16; participants were registered nurses n = 7, clinical nurse specialists n = 2, clinical nurse consultants n = 3, nurse unit manager n = 1, social workers n = 2, welfare officer n = 1 working within a substance use treatment setting sex: female (93.8%); age NR	Public health community-based substance use treatment services	
Hays et al. 2020; Mount Isa QLD	Rural and remote Australia	Quantitative cross-sectional	N = 19; participants were rural pharmacists; sex: female 63%; age < 25 n = 1; 26–35 n = 7; 36–45 n = 3; 46–55 n = 4; 56 + n = 4	Rural pharmacy services	
Henderson et al. 2018; Adelaide SA	Adelaide Hills (MM2), the Fleurieu Peninsula (MM3), and Kangaroo Island SA (MM7)	Qualitative interviews	N = 31; local service providers n = 25 and senior managers from major service providers n = 6; sex: NR; age NR	Service providers in mental health, community health, general practice, residential aged care, private practice, non-government organisations, and local government in the public and private sectors	
Hinton et al. 2015; Darwin NT	Remote NT	Qualitative interviews	N = 27; participant were NT government and local council representatives, education and early childhood service providers, employment and housing agencies, police and correctional services, alcohol and other drug (AOD) workers, remote health centre staff and Top End mental health staff; sex: NR; age NR	Indigenous mental health services	
Isaacs et al. 2017; Moe VIC	Echuca VIC (MM3)	Qualitative interviews	N = 27; participants were Aboriginal workers n = 24, senior mental health clinician n = 1, police officer n = 1, Aboriginal Elder n = 1; sex: female 44.4%; age > 18 years	Help seeking and suicide services for Aboriginal people in rural VIC	
Kidd et al. 2012; Unknown VIC	Rural VIC	Mixed methods: quantitative cross-sectional and qualitative focus groups	N = 17; participants were nurses working in rural EDs; sex: NR; age 73% ≥ 45 years, a small number were aged > 60 years, and one aged over 70 years	Nurses from rural health services who had ED clients in the previous 12 months	
Malatzky et al. 2020; Brisbane QLD	Large rural town	Qualitative interviews	N = 13; participants were allied health, medical, community, youth work, management and administration professionals; sex: NR; age NR	Mental health services for young people	
Mirza et al. 2019; location NR	Rural and remote Australia	Abstract of case reports	N = NR; participants were spiritual healers and Aboriginal mental health workers	General mental health services	
Mollah et al. 2018; Clayton VIC	Rural Australia	Mixed methods: quantitative cross-sectional and qualitative interviews	N = 20; participants had experience working with immigrant patients in the previous 12 months and were counsellors n = 4, age M = 45.25 years; psychologists/ psychiatrists n = 6, age years M = 37.5 years; nurses n = 5, M = 37.5 years; social workers n = 2, age M = 31.5 years; other n = 3, age M = 49.7 years	Mental health services for immigrant patients	
Muir-Cochrane et al. 2014; Adelaide SA	Rural Australia	Qualitative interviews	N = 19; participants were managers of residential and community aged care services, coordinators of programmes and care packages, nurses, OTs, social workers, counsellors, and mental health clinicians; sex NR; age NR	Older person’s mental health services	
Newman et al. 2016; Magill SA	Magill SA (MM1); regional, rural, remote SA areas serviced by Country Health SA local health network	Qualitative interviews and focus groups	N = > 40; participants were from a regional mental health team (i.e., managers, team leaders, senior clinicians, mental health NPs, administrative staff), the metropolitan mental health hub’s mental health team, and a tele-mental health support team; sex: NR; age NR	Telehealth mental health services	
Orlowski et al. 2016a; Adelaide SA	Rural SA	Qualitative interviews and focus groups	N = 48; interview participants were youth mental health clinicians n = 3, and support and management/executive staff n = 5; sex: female 50%; age: 18–40 years. Focus group participants were mental health and youth service teams, including social workers, mental health nurses, psychologists, psychiatrists, OTs, counsellors, youth workers, management, and other staff; sex: female 50–86% female; age NR	Technology-enhanced mental health services	
Orlowski et al. 2017; Bedford Park SA	Rural SA	Qualitative shadowing, non-participant observation, interviews, field noting, documenting analysis, debriefing	N = NR; participants at site 1 were youth workers n = 3, manager n = 1, clinical lead n = 1, medical staff n = 2, psychological staff n = 5, and other government staff; sex: NR; age NR; participants at site 2 were mental health nurses n = 7, social workers n = 3; sex: NR; age NR	Technology-enhanced mental health services	
Procter 2015; Adelaide SA	Rural SA	Qualitative focus groups	N = 9; participants were nurses n = 4, social workers n = 2, clinical psychologist n = 1, OT = 1; paramedical aid n = 1; sex: female 77.8%; age M = 46 years SD = NR	Community mental health services	
Taylor et al. 2019; Brisbane QLD	Regional, rural, and remote QLD	Qualitative interviews	N = 14; participants were medical officers, social workers, nurses, mental health clinicians, managers, and health promotion workers of an electronic perinatal and infant mental health service; sex: female 78.6%; age 26–62 years	Perinatal and infant tele-mental health services	
Trail et al. 2021; Parkville VIC	Macedon Ranges VIC (MM5)	Qualitative interviews	N = 19; participants were healthcare and health promotion professionals n = 8, community service/law enforcement/sports staff n = 8, educational staff n = 3; sex: female 52.6%; age M = 49.9; SD = 11.8	Healthcare and community services working in male suicide and harm prevention	
Wand et al. 2021a; Camperdown NSW	Maitland (MM1) and Dubbo (MM3) NSW	Qualitative interviews	N = 12; participants were MHLNs; sex: NR; age NR	ED-based mental health nursing care services	
***Service user perspective***					
Batterham et al. 2020; Canberra ACT	Metropolitan, regional, rural Australia	Quantitative cross-sectional	N = 2,374; participants from the Assessing Mental Health Survey [115] who met criteria for a DSM-5 mental disorder/suicidal ideation; sex: female 79.6%, 18–35 years n = 913, 36–55 years n = 878, 56 + years n = 582; metropolitan area (n = 1,249), regional area (n = 867), rural area (n = 258)	Perceived need for help for a mental health problem	
Black et al. 2012; Adelaide SA	Outside of metropolitan Adelaide, SA	Quantitative cross-sectional	N = 531; participants (school students) from the Adolescent Mental Health, Behaviour and Life Experiences Study (unpublished data; authors) who met criteria for a DSM-IV major depressive disorder; sex: female 55.7%; 13–18 years	General mental health services for rural adolescents with depression	
Butterfly Foundation 2020; location NR	Rural and remote Australia	Survey and report	N = 563; participants were individuals who currently or previously experienced an eating disorder, or were carers of people with eating disorders; sex: mainly female; age 18–60 years	Eating disorder services	
Byrne et al. 2017; Rockhampton QLD	Regional and rural areas QLD	Qualitative interviews	N = 13; sex: female 61.5%; participants were employees of peer-run, government, and non-government organisations	Peer workers working in mental health services in rural and regional locations	
Dawson et al. 2016; Adelaide SA	Rural SA	Qualitative interviews	N = 11; participants were rural carers of older people with a mental health issue; sex: NR; age NR	General mental health services	
Dunstan et al. 2014; Armidale NSW	Moree NSW (MM4)	Mixed methods: quantitative longitudinal and qualitative feedback comments	Total sample N = 76; sex: female 56.3%; age M = 37.8, SD = 13.0; Individual recovery plan completers n = 19; sex: female 42%; age M = 38.5, SD = 14.8; participants were current and past clients of the Personal Helpers and Mentors service (PHaMs), Moree, who had a diagnosed mental disorder	Personal Helpers and Mentors service in Moree	
Handley 2014; Callaghan NSW	Rural and remote NSW	Quantitative cross-sectional	N = 394; participants were from the 3-year follow-up of the Australian Rural Mental Health Study who had a self-reported mental health problem; help/advice sought: sex: female 56.0%; age M = 55.5 SD = 13.1; help/advice sought and needs met: sex: female 68.0%; age M = 55.6 SD = 13.1; help/advice sought and needs not met: sex: female 80.0%; age M = 55.9 SD = 12.1	Professional sources (e.g., GP, psychiatrist, psychologist, MH nurse, Lifeline, specialist doctor), and non-professional sources (e.g., family/friends, alternative therapist, clergy) for seeking mental health help	
Hussain et al. 2013; Armidale NSW	Armidale NSW (MM3)	Quantitative cross-sectional	N = 355; participants were full-time university students at a public university (the University of New England) in Armidale, NSW; sex: 69.0% females; age M = 20.2 SD = 4.8	Support services (e.g., counselling) and GP services	
Johnson et al. 2021; Wagga Wagga NSW	Wagga Wagga (MM3) and Riverina region, NSW (MM5)	Qualitative interviews	N = 27; participants were from a regional rural region in NSW who self-reported a mental health issue, some had a formal diagnosis and had accessed services and others had not accessed services; sex: female 100%; age ≥ 30 years, most in their 50s and some over 70 years	General mental health services for regional rural women with depression	
Orlowski et al. 2016b; Adelaide SA	Inner rural regions in SA	Qualitative interviews	N = 10; participants were a clinical sample of young people who were currently seeking help for a mental health issue; sex: female 50%; age 16–22 years	Technology-enhanced mental health services	
Reynish et al. 2021; Launceston TAS	Rural and remote TAS	Qualitative interviews	N = 6; participants were from a rural or remote TAS, who had compromised access to bodily autonomy (i.e., sex, sexual, gender diverse, or LGTBIQA + people, sex workers, people who are intersex, and kink-oriented people) and had a self-reported mental health issue; sex: female 66.7%; age 24–61 years	Mental health service use for sex workers	
Richardson et al. 2015; Murdoch WA	South West region WA (MM5)	Quantitative cross-sectional	N = 8; participants were engaged in a telepsychology psychotherapy service; sex: female 75.0%; age 27–52 years	Videoconferencing telepsychology services	
Wilson et al. 2012; Armidale NSW	Hunter New England region (MM3), NSW and Darling Downs, QLD (MM5)	Qualitative interviews	N = 13; participants had experienced of emergent mental health issues with symptoms of psychosis among men (either themselves or someone in their family); sex: 61.5% female; age 21–60 years	General mental health services	
***Combined health professional and service user perspectives***					
Bowman et al. 2020; Bathurst NSW	Rural, regional, remote Australia	Qualitative interviews	Group 1: n = 9; sex: female 22.2%, transgender 11.1%, non-binary 22.2%; aged 18–25 years. Group 2: n = 6; participants held roles of director n = 4, manager n = 1, and unspecified n = 1 within general mental health support, youth focused (12–25 years), issue-specific services (e.g., depression and anxiety), and lesbian, gay, bisexual, and transgender (LGBT)-dedicated support services; sex: NR; age NR	Users and providers of internet-based mental health services for LGBT young adults in rural areas	
Consumers of Mental Health WA 2018; Cannington WA	Rural WA	Submission to the senate	Participants were mental health service workers, Aboriginal or Torres Strait Islander people, individuals with a lived experienced of a mental health issue, and family members/friends of someone with a lived experienced of a mental health issue	General mental health services	
Henderson et al. 2014; Adelaide SA	Port Lincoln (MM6), Port Pirie (MM4), and Berri (MM5) SA	Evaluation; mixed methods: quantitative cross-sectional, qualitative interviews, mapping of client journey	N = 31; participants were key informants working within either the mental health team or external aged care agencies n = 22, clients n = 4, and carers n = 4; sex: NR; age NR	Older person’s mental health services	
Isaacs et al. 2012; Moe VIC	Gippsland, VIC (MM5)	Qualitative interviews and focus groups	N = 46; participants were Aboriginal men from the Community n = 12, Aboriginal carers of men diagnoses with a mental illness n = 2, Koori Hospital Liaison Officer n = 1; and social and emotional wellbeing workers n = 2; community mental health nurse n = 1; private psychologist n = 1; acute psychiatric care nurse n = 1; non-Aboriginal social and emotional wellbeing worker n = 1; emergency care liaison nurse n = 1; community mental health team members n = 24; sex: NR; age NR	Mental health services for Aboriginal men	
Isaacs et al. 2013; Moe VIC	Gippsland, VIC (MM5)	Qualitative interviews and focus groups	N = 17; participants were clients of mental health services n = 5, non-clients of mental health services n = 5, carers n = 2, cultural advisors n = 2; Aboriginal Hospital Liaison Officer n = 1, social and emotional wellbeing workers n = 2; sex: NR; age NR	Mental health services for Aboriginal men	
Mental Health Council of Tasmania 2018; Hobart TAS	Rural and remote Australia	Submission to the senate	N = NR; participants were consumers, carers, family members, service providers, and community members with involvement with the mental health sector	General mental health services	
Wand et al. 2021b; Camperdown NSW	Maitland (MM1) and Dubbo (MM3) NSW	Qualitative interviews	N = 60; participants were ED patients n = 32; nurses n = 14, ED medical officers n = 11, psychiatrists n = 3, patients n = NR; sex: NR; age: NR	ED-based mental health nursing care services	
Weber et al. 2012; Clunes NSW	Northern Rivers NSW (MM5)	Evaluation; mixed methods: quantitative cross-sectional, qualitative interviews	N = NR; participants were clients of an ED service and service providers including GPs, private practice psychologists and social workers, dietitians, and mental health service workers; sex: NR; age NR	Eating disorder services	
***Other***					
Bridgman et al. 2019; Hobart TAS	Hobart (MM2) & Glenorchy (MM2) TAS	Evaluation; quantitative longitudinal	Evaluation of Pulse Youth Health South—an outreach service consistent with headspace best practice guidelines; sex: NR; age 12–15 years	Mental health services for young people	
Duggan et al. 2020; Melbourne VIC	Regional and rural Australia	Report	Data were obtained from the Australian Institute of Health and Welfare [116]; sex: NR; age: NR	Mental health presentations in regional/rural emergency departments	
Knight et al. 2018; Mackay QLD	Regional QLD	Evaluation; quantitative longitudinal	Evaluation of the STARR model—an integrated care model between a regional adult mental health team and a non-government organisation; sex: NR; age: NR	Adult mental health services	
National Rural Health Alliance 2017; location NR	Regional, rural, and remote Australia	Fact sheet	Data were obtained from Mental Health Services in Australia [117]; sex: NR; age: NR	General mental health services	
Onnis et al. 2020; Cairns QLD	South of Mackay (MM2) to Cow Bay (MM6), and west of Croydon (MM7) and Richmond (MM7) QLD	Evaluation; quantitative longitudinal	Evaluation of Connect To Wellbeing—an initial assessment and referral service to improve psychological service access for people on low incomes; sex: NR; age: NR	Mental health intake and assessment service	
Salinas-Perez et al. 2020; Kimberly Region, WA	Remote WA	Long-term care service description and classification	Information on service provision was gathered from managers of local organisations and through mental health atlases; sex: NR; age: NR	Mental health services in the Kimberly Region, WA	
van Spijker et al. 2019; Western NSW and Country WA local health districts	Rural western NSW and WA	Long-term care service description and classification	Information on service provision was ascertained from peak bodies and sector representatives in Primary Health Networks (PHNs); sex: NR; age: NR	Mental health services in Western NSW and Country WA PHNs	

*Note: NR = not reported; ACT = Australian Capital Territory; NSW = New South Wales, NT = Northern Territory, QLD = Queensland, SA = South Australia, TAS = Tasmania, VIC = Victoria, WA = Western Australia; AOD = alcohol and other drugs; CALD = culturally and linguistically diverse; DSM = Diagnostic and Statistical Manual of Mental Disorders; ED = emergency department; GP = general practitioner; MHLN = mental health liaison nurse; MM = Modified Monash Model; NP = nurse practitioner; PHaMs = Personal Helpers and Mentors service; PHN = Primary Health Network; NDIS = National Disability Insurance Scheme*

### Geographical analysis

Geographical coordinates provided by the health workforce locator [26] were used to determine the remoteness of the study locations according to the MMM categories. These data were inputted into STATA to determine the number and proportion of each of the MMM categories.

## Results

**Fig. 1:**
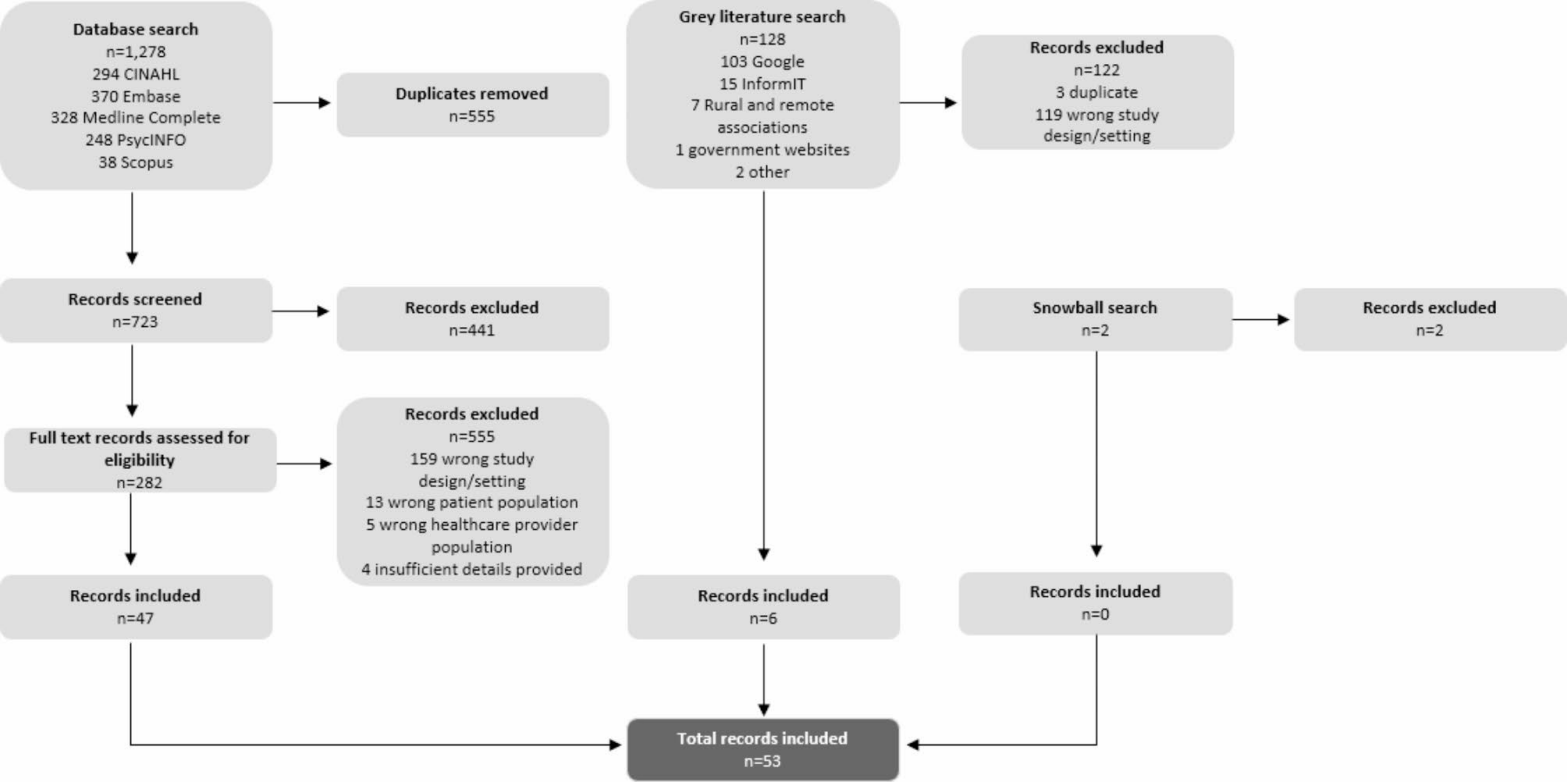
PRISMA flow diagram of studies considered in this review

The database search yielded 1,278 articles, of which 555 articles were removed due to duplication. Subsequently, 723 titles and abstracts were screened, and 441 were excluded due to ineligibility. At the full text stage, 282 articles were screened, with 181 studies being excluded, resulting in 47 articles meeting the eligibility criteria. The grey literature search yielded 128 potentially relevant sources, of which six were eligible after removing three for duplication. In total, 53 articles were included in this scoping review. A snowball search of the references of included records was also conducted and two additional records were identified but were deemed ineligible as they reported on studies/samples that were already included in the review. Figure 1 displays the PRISMA flow throughout each screening stage.

### Study characteristics

Of the 53 included studies, 25 articles described barriers and/or facilitators from the healthcare provider perspective, 13 were from the point of view of the service user, eight reported on combined perspectives of both the healthcare provider and service user, and seven reported on barriers/facilitators from neither the healthcare provider nor service user perspective directly but did consider the barriers/facilitators of the service environment (e.g., service evaluations).

Most studies (n = 29, 54.7%) employed qualitative methods, including interviews and/or focus groups; 12 studies utilised quantitative cross-sectional or longitudinal methods, seven were mixed-methods research designs, and two were service description and classification studies.

The highest proportion of studies were conducted in New South Wales (NSW) (n = 13) [31–43], followed by Australia broadly (n = 12) [33, 44–54], South Australia (SA) (n = 10) [55–64], Victoria (VIC) (n = 6) [65–70], Queensland (QLD) (n = 5) [71–75], Western Australia (WA) (n = 3) [76–78], Tasmania (TAS) (n = 2) [79, 80], and Northern Territory (NT) (n = 1) [81]. One study pertained to areas within NSW, QLD, and VIC [82], and another study concerned NSW and WA [83]. No studies were centred on Australian Capital Territory (ACT). Table 2 depicts the characteristics of the included studies.

### Setting and target groups

#### Mental health service setting

Fourteen studies reported on general or community-based mental health services [18, 33, 43, 48, 53, 54, 57, 64, 72, 74, 77, 78, 83]. Four studies described mental health services provided within emergency departments (EDs) and/or urgent care centres (UCCs) [40, 41, 46, 65]. The remaining studies described mental health services provided by counsellors and GPs [38], nurses, peer-workers [71], personal helpers and mentors [35], pharmacists [47], and a combination of several healthcare providers [59]. Seven studies reported on technology-based or -enhanced mental health services [51, 60–63, 75, 76].

#### Target groups

The population group focus of studies varied. Of the studies that commented on, or specified that they targeted specific subpopulations, four studies discussed care pertinent to Indigenous or Aboriginal and/or Torres Strait Islander Peoples [66–68, 81]. Four studies discussed mental health services for young people [55, 63, 73, 82]. Three studies specifically included at least a proportion of service users who were under the age of 18 years old [55, 61, 79]. Two studies reported on mental health services for older people [50, 58]. Other studies described barriers and or facilitators specific to sex workers [80], medical doctors [45], LGBTIQA + people [51], immigrants [49], and women [39] or men [70] with specific mental health issues. Three studies described mental health services that were specific for supporting people with depression [34, 39, 55]; two studies were focussed on suicide [68, 70]; two studies described care for people with eating disorders [42, 52]; and one study was centred on perinatal and infant support [75].

### Barriers and facilitators

The included studies varied significantly. This included differences in the purpose and type of study, participant sample, and methodology, and reporting of findings. Barriers and facilitators were grouped into prominent concepts based on terminology used by the relevant literature and are presented in Table 3. Barriers related to limited resources; system complexity and navigation; attitudinal and social matters; technological limitations; distance to services; insufficient culturally-sensitive practice; and lack of awareness. Facilitators related to person-centred and collaborative care; technological facilitation; environment and ease of access; community supports; mental health literacy; and culturally-sensitive practice.

**Table 3:** Key concepts across the included studies

Key concept	Example/s of specific issues related to key finding area	Citation relevant to key finding area*
***Barriers***		
***Barriers affecting healthcare providers and service users***		
Limited resources	• Lack of available general and specialist services • Limited service capacity • Workforce shortages • Difficulty attracting and retaining staff • High turnover of staff • Time constraints • Limited funding • Financial disadvantage • Costs for service users • Lack of transport • High workloads	Beks et al. [65], Cosgrave et al. [18], Cosgrave et al. [33], De Silva et al. [34], Dunstan et al. [35], Evans et al. [36], Hussain et al. [38], Johnson et al. [39], Clough et al. [45], Muir-Cochrane et al. [50], Bowman et al. [51], Butterfly Foundation [52], Mental Health Council of Tasmania [53], National Rural Health Alliance [54], Crotty et al. [56], Henderson et al. [58], Henderson et al. [59], Newman et al. [60], Orlowski et al. [61], Orlowski et al. [62], Orlowski et al. [63], Procter et al. [64], Isaacs et al. [66], Byrne et al. [71], Onnis et al. [74], Taylor et al. [75], Consumers of Mental Health WA [78], Bridgman et al. [79], Reynish et al. [80], Ellem et al. [82], Black et al. [55], Weber and Davis [42]
System complexity and navigation	• Long waiting times • Inefficient referral pathways • Lack of care-coordination • Delays in assessment and diagnosis • Difficulty navigating services • Limited training • Professional culture or organisational functions	Evans et al. [36], Clough et al. [45], Butterfly Foundation [52], Beks et al. [65], Cosgrave et al. [33] Cosgrave et al. [18], Henderson et al. [59], Kidd et al. [69], Malatzky et al. [73], Orlowski et al. [63], Wand et al. [41], Bowman et al. [51], Dawson et al. [57], Orlowski et al. [61], Reynish et al. [80], Consumers of Mental Health WA [78], Henderson et al. [58], Isaacs et al. [66], Mental Health Council of Tasmania [53], Wand et al. [40], Weber and Davis [42], Duggan et al. [46], Onnis et al. [74]
Attitudinal and social matters	• Stereotypical views of patients • Stigma • Fear of judgement • Lack of trust or confidence in services • Privacy and confidentiality concerns • Consumer vulnerability • Preference for keeping to oneself • Stoicism • Reluctance to seek help • Lack of awareness about mental health issues • Dual-relationships	Butterfly Foundation [52], Hinton et al. [81], Muir-Cochrane et al. [50], Procter et al. [64], Trail et al. [70], Dunstan et al. [35], Evans et al. [36], Hussain et al. [38], Johnson et al. [39], Orlowski et al. [61], Reynish et al. (72), Wilson et al. [43], Bowman et al. (31), Consumers of Mental Health WA [78], Isaacs et al. [66], Isaacs et al. [67], Isaacs et al. [68], De Silva et al. [34], [36], Newman et al. [60]
Technological limitations	• Poor connectivity • High cost • Lack of suitability for use among specific client groups	Bowman et al. (31), Orlowski et al. [62], Orlowski et al. [61], Procter et al. (56), Consumers of Mental Health WA [78], Mental Health Council of Tasmania [53], Newman et al. [60]
Lack of awareness	• Lack of awareness about mental health issues • Lack of awareness of needs • Lack of awareness about services available	Wilson et al. [43], Muir-Cochrane et al. [50], Butterfly Foundation [52], Isaacs et al. [67], Consumers of Mental Health WA [78]
***Barriers affecting service users***		
Distance to services	• Lengthy travel time due to distance to services	Butterfly Foundation [52], Orlowski et al. [62], Orlowski et al. [63], Byrne et al. [71], Consumers of Mental Health WA [78], Mental Health Council of Tasmania [53], Procter et al. [64]
Insufficient culturally-sensitive practices	• Lack of culturally-suited staff, processes, and services • Cultural assumptions about experience and medical treatment	Malatzky et al. [73], Mirza [48], Mollah et al. [49], Consumers of Mental Health WA [78], Henderson et al. [58]
***Facilitators***		
***Facilitators affecting healthcare providers and service users***		
Person-centred and collaborative care	• Interservice collaborations • Shared knowledge • Informal working relationships • A non-judgemental and positive approach to service delivery • Involving clients and their families in care • Regular contact • Continuity of care • Appropriate and skilled communication • Flexibility in meeting places	Barraclough et al. [31], Beks et al. [65], Crotty et al. [56], De Silva et al. [34], Evans et al. [36], Henderson et al. [59], Orlowski et al. [63], Hinton et al. [81], Procter et al. [64], Wand et al. [41], Dawson et al. [57], Dunstan et al. [35], Orlowski et al. [61], Henderson et al. [58], Weber and Davis [42], Knight et al. [72], Onnis et al. [74]
Technological facilitation	• Technology used in conjunction to face-to-face service delivery • SMS appointment reminders	Newman et al. [60], Orlowski et al. [62], Orlowski et al. [63], Taylor et al. [75], Orlowski et al. [61], Richardson et al. [76], Bowman et al. [51], Consumers of Mental Health WA [78], Henderson et al. [58], Mental Health Council of Tasmania [53]
Environment and ease of service access	• Non-clinical and comfortable environment • Easy access to after-hours services • Services delivered by dedicated staff with local knowledge • Screening for specific disorders if supported by organisational tools • Outreach options • Co-location of services • Organisational culture, priorities, systems, and structures • Low/no cost services	Barraclough et al. [31], Beks et al. [65], Hinton et al. [81], Malatzky et al. [73], Mollah et al. [49], Reynish et al. [80]
Community supports	• Sense of community • Clinicians being involved in the community and knowing local issues • Clinician visibility outside of health-provider role	Crotty et al. [56], Henderson et al. [59], Johnson et al. [39], Beks et al. [65], Isaacs et al. [66]
***Facilitators affecting service users***		
Mental health literacy	• Familiarity and confidence in using services • Knowledge of mental health services	Butterfly Foundation [52], Henderson et al. [59], Trail et al. [70], Dawson et al. [57], Isaacs et al. [66]
Culturally-sensitive practice	• Cultural competency • Use of Aboriginal mental health workers, spiritual healers, and involvement of the community elders	Mirza [48], Mollah et al. [49], Isaacs et al. [66]

Note: *Citation relevant to overall concept

#### Prominent barrier concepts

##### Barriers affecting healthcare providers and service users

***Limited resources.*** Across the studies, the most considerable barrier was limited resources [18, 33–36, 38, 39, 42, 45, 50–56, 58–66, 71, 74, 75, 78–80, 82]. This key concept considered limited resources at the healthcare provider and service user level. Notably, lack of available general and specialist services, limited service capacity, workforce shortages, difficulty attracting and retaining staff, and staff turnover were frequently reported as considerable spatial barriers to service delivery, hampering access to services. Moreover, financial costs, disadvantage, or appointment fees [34, 37, 52, 53, 61, 62, 78], and lack of transport [34, 50, 52, 53, 58, 62, 71, 78] restricted access to mental health services for the service user. These issues reflect the lower relative socio-economic advantage seen in rural areas of Australia [2].

***System complexity and navigation.*** The complexity in using and navigating the system was a common aspatial barrier [18, 33, 36, 40–42, 45, 46, 51–53, 57–59, 61, 63, 65, 66, 69, 73, 74, 78, 80], which affected healthcare providers in coordinating patient care and service users in utilising such care. These issues were most frequently reflected in reports on extended wait times and delays in assessment and diagnosis [34, 40, 46, 53, 55, 57, 58, 62, 66, 78, 80].

***Attitudinal or social matters.*** Many studies reported that attitudinal or social matters were a barrier for the service user [34–36, 38, 39, 43, 50–52, 60, 61, 64, 66–68, 70, 78, 80, 81], particularily concerning privacy or confidentiality concerns [39, 51, 60–63, 66, 67, 78], affecting aspatial access to care. The need to be stoic was reported as a barrier to seeking psychological help among regional medical doctors, relating to their perceptions of regional practitioner identity [45], and among service users [50, 67, 70].

***Technological limitations.*** Several studies cited limitations to services delivered via technological means [51, 53, 60–62, 64, 78]. Some studies acknowledged that technology can enhance physical mental health services, but cannot replace them [62, 64], particularly for specific client groups, including the older population and Aboriginal and Torres Strait Islander people, who reportedly prefer face-to-face service delivery [78]. In addition, poor connectivity and high costs of technology use were reported as aspatial barriers to accessing technology-delivered mental health services and may also affect their utilisation [53, 62, 78].

***Lack of awareness.*** Lack of awareness about mental health issues, needs, or services available was reported as an aspatial barrier in the current review [43, 50, 52, 67, 78]. This lack of awareness was reported at the healthcare provider level in one study, and was described as the healthcare provider having a limited understanding of the mental health needs in older people, resulting in a lack of referral to appropriate services [50]. At the service user level, a lack of awareness precluded individuals from recognising mental health problems [67], while a lack of awareness of services was a barrier to seeking help [52, 78].

##### Barriers affecting service users

***Distance to services.*** The spatial distance required to travel to physical services is a considerable issue for people residing in rural localities, and this distance has been shown to reduce service access and utilisation in the current review [52, 62–64, 67, 71, 78]. There is also an additional burden experienced by those with physical disability, or those who don’t have a support person to assist them [53].

***Insufficient culturally-sensitive practice.*** A limited capacity to meet the needs of culturally and linguistically diverse (CALD) and Aboriginal and Torres Strait Islander communities was reported, affecting aspatial access and utilisation of services. This tended to be a result of service users not feeling culturally safe within the service environment, perceptions that health professionals had cultural assumptions about the service user, and inappropriate assessment tools [48, 49, 58, 73, 78].

#### Prominent facilitator concepts

***Facilitators affecting healthcare providers and service users***.

***Person-centred and collaborative care.*** Many studies reported that person- (or client-) centred care that is non-judgemental and permits collaboration to be an important aspatial facilitator to mental health service access and utilisation [31, 34–36, 41, 42, 56–59, 61, 63–65, 72, 74, 81]. It is noteworthy that person centred care was specifically reported in studies pertaining to the service user [61] and healthcare provider [63, 64] in the current review, suggesting that this approach is recognised as important by both those delivering and using the service. Care that is regular and non-intrusive was seen as a way to facilitate service utilisation [34, 57].

***Technological facilitation.*** Technology-based services, including integrated mental health services, telehealth, live chat, SMS appointment reminders and coordination, and mental health web-pages, were reported to be useful in filling spatial and aspatial gaps in service delivery for physical services [51, 53, 58, 60–63, 75, 76, 78]. These services were reported to facilitate connection and information sharing [62], clinical supervision, contact with specialists [60], workforce upskilling, and security [75] for the healthcare provider. For the service user, technology-based services facilitated immediacy of consultations, cost savings, and anonymity, and reduced mental health hospitalisations and admissions, additional client appointments, the need to travel, stigma, and family stress [60].

***Environment and ease of access.*** The mental health service environment and the ease of which one may access services — granted that all other access issues are overcome — were frequently reported as spatial facilitators [31, 49, 65, 73, 80, 81]. Specifically, services that permitted a non-clinical and comfortable environment were deemed as important aspatial factors for young people [61, 73]. Co-located services were also considered important for access, as this allows service integration and facilitated information sharing [31, 41, 63].

***Community supports.*** The community was considered to be an important aspatial facilitator. This included healthcare providers being involved and connected with the community [56, 65, 66], as well as having a sense of community [59], as a way to facilitate care via information sharing, collaboration, and knowing community members and local issues. For the service user, community and place was seen as a source of strength as noted by one study [39].

##### Facilitators affecting service users

***Mental health literacy.*** Several studies reported that having awareness of mental health issues and being confident in using services were aspatial facilitators to mental health service access and utilisation [52, 57, 59, 66, 70]. These factors are generally referred to as mental health literacy within the wider literature, which is a crucial component of healthcare [84].

***Culturally-sensitive practice.*** Of the studies that reported on cultural elements of mental health service provision, it was noted that Indigenous and other culturally appropriate staff (i.e., a Koori Mental Health Liaison Officer or Aboriginal Mental Health Worker), as well as the involvement of Community Elders and spiritual healers [48] assisted with service access and utilisation [48, 66]. Further, culturally appropriate décor and flexibility in meeting places [66], and the use of culturally acceptable models of mental health [48] were also seen as important aspatial dimensions.

### Geographical analysis

Overall, thirty studies were described as being relevant to rural areas [18, 31, 33–36, 38, 39, 42, 43, 48–50, 53, 57, 58, 61–70, 73, 76, 78, 83], three studies were pertinent to regional areas [39, 56, 79], two studies were concerned with remote areas [77, 81], and the remaining studies involved combinations of regional/rural/remote populations of Australia [37, 40, 41, 44–47, 51, 52, 54, 55, 59, 60, 71, 74, 75, 80, 82]. Over one third of the studies (n = 21, 39.6%) reported or provided specific spatial data, which allowed the MMM [4] to be applied directly to the study location; n = 10 (47.6%) of these studies included multiple locations, resulting in a total of 41 MMM categories. Studies were conducted most frequently in *MM5 small rural towns* (n = 10, 24.4%) and *MM3 large rural towns* (n = 9, 22.0%) and least frequently in *MM6 remote communities* (n = 3, 7.3%). The first author’s location was used as a proxy location for 28 studies (52.8%). Of these studies, the most frequent location was *MM1 metropolitan settings* (n = 16, 57.1%), likely due to the high proportion of study locations being taken from the first author’s location, and that many universities and research centres are located in major cities. There were no studies conducted in *MM5 small rural towns* (n = 0, 0%). Three author locations (5.7%) could not be determined due to limited information provided. Table 4 displays details of the MMM categories according to spatial data reported or obtained and proxy locations. Due to the heterogeneity and lack of mutual exclusivity of the data, an analysis of the association between geographical area and specific barriers and facilitators was unable to be completed.

**Table 4:** MMM categories according to spatial data reported or obtained and proxy locations

	Spatial data reported or obtained		Proxy location used	
	*n*	%	*n*	%
MM1*	4	9.8	16	57.1
MM2	5	12.2	5	17.9
MM3	9	22.0	4	14.3
MM4	6	14.6	1	3.6
MM5	10	24.4	0	0.0
MM6	3	7.3	1	3.6
MM7	4	9.8	1	3.6
Total	41	100%	28	100%

Note: MMM = Modified Monash Model; *Studies pertaining to MM1 areas are excluded from this review, with the exception of the included studies that described the setting as regional/rural/remote but were classified as metropolitan when the MMM was applied. Data are not mutually exclusive

## Discussion and implications

This scoping review identified the barriers and facilitators experienced by healthcare providers delivering mental health services and individuals accessing, or attempting to access mental health services in rural Australia. Prominent barriers included: limited resources; system complexity and navigation; attitudinal and social matters; technological limitations; distance to services; insufficient culturally-sensitive practice; and lack of awareness. Facilitators included person-centred and collaborative care; technological facilitation; environment and ease of access; community supports; mental health literacy and culturally-sensitive practice. We also aimed to understand these barriers and facilitators in relation to their geographical context; however, the variability in the data precluded the geographical analysis from being completed.

This study revealed a paucity of research conducted in *MM6 remote* and *MM7 very remote communities* in Australia when specific spatial data are considered, as well as in the ACT — however, it is noted that the majority of the ACT is classified as metropolitan, with 99.83% (387,887 residents) of the population residing in MM1 at the time of the 2016 census [2]. Moreover, when proxy study locations are used, many studies are conducted by researchers located in metropolitan areas. Only three studies specifically included service users who were under the age of 18 years old, representing a significant gap in understanding the mental health service needs of the younger population. Although it is acknowledged that there are considerable research ethics restrictions in place to protect children and young people, the onset of many mental health issues tends to occur between 14.5 and 18 years of age [85], highlighting the importance of understanding barriers and facilitators to accessing mental health services amongst the younger cohort. Due to the heterogeneity of the findings, the following discussion considers the most prominent barriers and facilitator concepts identified across the studies.

Review findings support limited resources as being one of the biggest restrictors of mental health service access and utilisation within rural Australia. Thes findings echo reports at the national scale, which show the mental health workforce is heavily concentrated in metropolitan areas compared to other remoteness areas, relative to the population [86]. Considerable efforts need to be made to reduce the resource inequalities, including the dearth of mental health professionals practicing outside of metropolitan cities. Recently, the National Mental Health Workforce Strategy Taskforce (the Strategy) was established to deliberate the quality, supply, distribution, structure, and methods to improve attracting, training, and retaining Australia’s mental health workforce [87]. The Consultation Draft of the Strategy highlights six objectives, including *(1)* careers in mental health are recognised as, attractive; *(2)* data underpins workforce planning; *(3)* the entire mental health workforce is utilised; *(4)* the mental health workforce is appropriately skilled; *(5)* the mental health workforce is retained in the sector; and *(6)* the mental health workforce is distributed to deliver support and treatment when and where consumers need it [88]. These objectives reflect the systemic resource issues cited in the current scoping review and emphasise the importance of a contemporary approach to increasing resources for mental health services in rural Australia. This contemporary approach is important, as it has previously been acknowledged that increasing graduates has not resolved workforce maldistribution in other areas of healthcare (i.e., medical physicians), but rather, an improved distribution of both human and other resources is needed [89, 90].

For the service user, resource issues spanned both aspatial and spatial dimensions and include the affordability (i.e., perceived worth relative to cost) and accessibility of the service (i.e., the location of the service and ease of getting to that location) [12, 13]. Transport issues were commonly reported to be a resource issue within the current review and the wider literature. Limited transport compounds access issues for specific subpopulations, such the elderly, particularly when they do not have personal transport and when there is no public transport available [50]. This issue is likely compounded by resource limitations, including the cost of travel, and is specifically related to spatial distance to services. Distance to services is a significant barrier to accessing healthcare. Wood et al. [91] in a systematic review, identified that there is a lack of research which measures spatial access specific to mental health services in Australia, and highlighted a need for consensus on what is reasonable access to healthcare services. Further, reports have noted that while distance alone is a significant barrier to accessing healthcare, accommodation may sometimes need to be sought depending on the time of the appointment, adding to the cost of attending the appointment [92] and further perpetuating the resource issues experienced by those living in rural areas of Australia. In addition, although not specifically reported in the current review, it is likely that the time required for traveling to and attending such appointments may require the individual to choose between tending to work or family needs or receiving the help needed.

Transport and other resource issues, as well as distance to services, may be mitigated through telehealth appointments, which have been central to the provision of healthcare since the beginning of the COVID-19 pandemic. However, the utilisation of telehealth requires many patients to have had a face-to-face consultation with their GP in the previous 12 months [93], which may preclude some Australians from rural areas from its use, considering the significant workforce maldistribution previously discussed. Moreover, rural areas of Australia also experience digital disadvantage as a result of lower internet connectivity — brought about by the high costs of installing internet infrastructure in rural and remote areas — and the socio-economic disadvantage experienced by those who live outside of metropolitan areas [94]. These issues are compounded by an ageing population, lower educational levels, a larger primary industry sector, a higher unemployment rate, and a higher Indigenous population in rural and remote Australia [94]. High cost, connectivity issues, and suitability for specific client groups should be key considerations in the delivery of technology-based mental health services. Notwithstanding these issues, the current review identified that technology-based services may be a useful adjunct to physical services, particularly in relation to reducing the need to travel, consultation immediacy, and clinician upskilling. This finding partially supports a recent systematic review, which found that youth located in rural and remote areas of Australia and Canada prefer to see mental health professionals in person, with telehealth provided as an additional option [95]. As such, the benefits and limitations to technology-based mental health services needs to be carefully considered by those designing services.

A key barrier to both access and utilisation in the current review was the complexity of using and navigating the mental health system. These issues typically occur at the system and organisation level and affect the way a service operates and its culture, making it challenging for service users to receive effective care. A complex mental health system and service fragmentation has been previously reported to lead to confusion and a lengthy amount time spent trying to navigate the system, with these issues being even greater amongst those who are younger, less autonomous, or who have less experience navigating the system [96]. System navigation initiatives may address this gap and have previously been implemented via the Partners in Recovery (PIR) program — established to facilitate care coordination for people with severe and persistent mental illness — with positive impacts for those who used the program [97]. However, the introduction of the National Disability Insurance Scheme has superseded the PIR program, and has rendered many former PIR program participants ineligible for support [98, 99], representing a significant gap in mental health service navigation and care coordination support. Isaacs et al. [100], identified that it is more cost effective to support people with severe and persistent mental illness to access PIR supports than to not provide this support, due to the potential increased need for other services (e.g., hospital admissions, homelessness supports, residential supports). Indeed, the Australian Government’s Productivity Commission (Productivity Commission) recommended that life insurers should have greater flexibility to fund approved mental health services to reduce the likelihood of hospitalisation for mental health issues [101]. In addition, Isaacs et al. [100] reported that co-located services — which were reported as a facilitator in the current review — and the increased need of non-clinical support through mental health community support services, offered via non-governmental and not-for-profit organisations, were demonstrated to be important considerations for cost effective mental health care.

Attitudinal or social matters are frequently reported to be key barriers for rural Australians to accessing care and are considered to be an aspatial dimension [12, 13]. These matters which include stigma, fear of judgement, stoicism, lack of trust, preference for keeping to oneself, and reluctance to seek help have been reported on the global scale as impacting upon help-seeking in rural areas in relation to rural identity [22]. Stoicism, in particular, is ordinarily viewed as a positive trait, with rural participants of a global review contextualising stoicism as an inflexible element to their core identity, however, this trait has repeatedly been reported as a barrier to the uptake of mental health services in this review [45, 50, 67, 70] and in the wider literature [22]. In terms of addressing attitudinal and social matters, previous Australian research [16] has identified that intentions to seek help for a mental or emotional issue decreased with a higher classification of remoteness. Moreover, stoicism and attitudes towards seeking professional help were predictive of help-seeking intentions for participants from both rural and metropolitan areas, but sex, suicidality, and previous engagement with a mental health professional were additionally predictive of help-seeking intentions for rural Australians [16]. The current scoping review identified few studies that specifically reported on these issues in relation to barriers to accessing services [37, 55, 68, 70], suggesting a need to increase research focus on these issues. Interestingly, Kaukiainen and Kõlves [16] study, found that attitudes towards seeking professional help mediated the relationship between stoicism and help-seeking intentions for participants from both rural and metropolitan locations, suggesting that attitudes towards seeking professional help may be a fruitful avenue to target to increase help-seeking intentions for all Australians [16]. Education programs delivered in secondary school or tertiary settings have been suggested as a way to improve attitudes towards help-seeking and stigma [102]. These avenues may also be useful to increase mental health literacy (i.e., the public knowledge and recognition of mental disorders and knowing where and how to seek help) [84] in the community, given that lack of awareness was a barrier and mental health literacy was a facilitator in the current review.

Providing person-centred and collaborative care was reported as a key facilitator in the current review. Person-centred care is generally defined as care that is holistic and incorporates the person’s context, individual expression, beliefs, and preferences, and includes families and caregivers, as well as prevention and promotion activities [103]. Indeed, person-centred care is a prominent practice model in mental health care, and this model of care may be particularly beneficial in rural Australia, given that it aims to decrease barriers between health service providers via shared knowledge. This model of care is collaborative by nature, although it should be noted that collaborative care is a distinct, though related model of care. Collaborative care refers to health professionals and patients working together to overcome a mental health problem [104]. This model of care has been shown to improve depression and anxiety outcomes across the short to long term (i.e., 0–24 months), and has benefits on medication use, patient satisfaction, and mental health quality of life [104]. The Productivity Commission recommended the trial of innovation funds to diffuse best practice in mental health service delivery and to eliminate practices that are no longer supported by evidence [101]. Such innovation funds may allow healthcare providers to maintain currency on practices such as person-centred and collaborative care. Importantly, the Royal Commission into Victoria’s Mental Health System (the Royal Commission) [90] identified person-centred care as a way to promote inclusion and prevent inequalities, and was specifically linked to providing culturally safe mental health care — which was noted as a facilitator to access and utilisation in the current review and has been highlighted as an important approach to eliminate health inequalities [105]. Moreover, the Royal Commission recommended the use of an integrated service approach — where service providers can work together to provide care [90]. This approach to care may mitigate service fragmentation and system complexity and navigation barriers, and also permit environments that are comfortable and allow ease of use — as identified as facilitators in the current review.

Community support, both in the sense of individuals feeling connected to the community and healthcare providers being seen within the community, was a key concept in the current review. For the service user, Johnson et al. [39] reported that accessing services under the scrutiny of the community was seen as a challenge, but that the community was also seen a source of strength. Crotty et al. [56] noted the duality for healthcare providers being involved with the community in both a social and professional sense, leading to both challenges and a feeling of togetherness. This sense of togetherness reflects the historical view that rural and remote communities have been connected over several generations [106]. Notably, in the current review, one study on healthcare provider perspectives on workforce retention reported that personal connections and a ‘natural’ connection to the community were key factors in the decision for staff working in remote areas to stay [33], suggesting the importance of embedded relationships in this setting. Preferences to stay in rural and remote towns have been associated with a sense of belonging and the quality of diverse and interesting activities, particularly for younger people [107], and these factors should be strengthened to permit the retention of the rural mental health workforce.

It is noteworthy that many of the studies were undertaken at metropolitan locations, suggesting that much of the research completed on rural locations was not necessarily conducted within this setting. However, it is acknowledged that many university locations are affiliated with major campuses, which are often located in metropolitan areas. Simultaneously, many rurally-based health and community services do not have the resources to undertake locally-generated research, and this consequently limits the evidence available for policymakers to make informed decisions regarding the health of the rural population — noting that place-based approaches are gaining traction [108–110]. This area is a key focus of the RHMT program [111]. The RHMT program aims to maximise investment in of Australia via academic networks, developing an evidence-base, and providing training in rural areas for health professionals. To date the RHMT program has seen that health graduates who undertook clinical placements in the most rural settings are working more in rural locations [112], and this is likely to have flow-on effects for healthcare providers to build connections to these areas, retain the workforce, and increase health outcomes for the community.

This review highlights the need for a contemporary approach to mental health services in rural Australia. This includes encouraging and educating the public about mental health issues and how to seek and engage in timely mental health care that is appropriate to one’s needs. Simultaneously, this review suggests a need to reconsider how the public navigates mental health services, and to redesign services that are easy to engage with, culturally safe, comfortable to use, and have technological capabilities. This may be more accurately achieved when services are designed with local issues and the community in mind via the integration of bottom-up place-based strategies and top-down place-sensitive approaches, particularly given that a one-size-fits-all approach to policy — and thus mental health service design — does not favour regions and localities [113]. It is critical that rural mental health services are invested in to remove barriers and improve health equity. The fiscal implications of such investment may be offset using this integrated approach, which leverages local and external assets, encourages workforce retention, and may reduce costs in other areas healthcare service delivery.

### Strengths and limitations

The strengths of this scoping review include the use of peer-reviewed and grey literature, the full-span of the child-adult age range, and the wide variety of included studies. In addition, this scoping review applied a consistent approach to applying remoteness categories, albeit this application was not without issues. For example, Wand et al. [40] and Wand et al. [41] reports on work done in Maitland (MM1) and Dubbo (MM3). Maitland (NSW) is of particular interest in the context of remoteness settings as it has historically been described as a regional area. In the early 2000s when the Australian Bureau of Statistics was defining the most accessible category of the Accessibility/Remoteness Index of Australia (ARIA), Maitland (as well as other locations such as Wollongong, NSW and Geelong, Victoria) was included in the most accessible category [114].

Several limitations must also be considered. Firstly, many sources — particularly grey literature sources — included potentially relevant information; however, a lack of clear evidence that the data specifically pertained to those living in regional/rural/remote areas prevented many of these sources from being included. In addition, findings were limited by the available literature, especially among community service organisations, which have limited resources to generate research outputs. The search strategy was limited to 2012–2022 and did not include search terms specific to certain subgroups of the population who have been known to experience barriers to mental health services in rural areas (e.g., farmers and people from CALD backgrounds), and some search results may have been omitted as a result of this. It was not possible to discern whether findings related specifically to access or utilisation in many studies, and as such, a nuanced discussion of these dimensions is not provided. Further, the data were heterogeneous and results tended to be grouped across regional, rural, and/or remote contexts, precluding an analysis of the association between geographical area and barriers and facilitators from taking place. Future research may consider completing a comprehensive geographical analysis once additional data on the topic becomes available. Lastly, although data screening was completed by two reviewers, only one reviewer coded the extracted data into key concepts, and this may have introduced bias into the results, however the key concepts were agreed upon by the research team.

### Conclusion

This scoping review found a number of barriers to accessing and utilising mental health services that may be overcome through initiatives that have been implemented or suggested by the government. Importantly, many of the spatial barriers associated with access and utilisation may be mitigated through innovative solutions, such as a combination of face-to-face and technology-based service provision, provided that careful consideration is given to the technological and resource limitations seen in the rural context of Australia. Parallel with this, several facilitators to accessing and utilising mental health services were noted, some of which may already be prominent in the provision of services, but could be further strengthened through additional training, service re-design, and community initiatives.

The included studies varied in their aim, setting, and study design, and many studies were grouped across MMM categories, disallowing a nuanced understanding of how barriers and facilitators operate within specific geographical contexts. This, paired with the finding that many studies were conducted at a metropolitan location, highlights the importance of conducting research within the rural setting. Additional research generated from rural areas, as well as consideration for how remoteness is measured, would assist in providing a more comprehensive understanding of the barriers and facilitators to mental health services within the geographic contexts they occur.

### Electronic supplementary material

Below is the link to the electronic supplementary material.

Additional Table 1: Search strategy for Medline Complete via EBSCO

Additional Table 2: Charting form used for data extraction

Additional Table 3: Barriers and/or facilitators of access and/or utilisation factors in regional, rural, and remote Australia

## Data Availability

The datasets used and/or analysed during the current study are available from the corresponding author on reasonable request.

## List of Abbreviations

ACT: Australian Capital Territory
ARIA: Accessibility/Remoteness Index of Australia
CALD: Culturally and linguistically diverse
CINAHL: Cumulative Index of Nursing and Allied Health Literature
ED: Emergency department
FTE: Full time equivalent
GP: General practitioner
LGBTIQA +: Lesbian, gay, bisexual, transgender, intersex, queer/questioning, asexual
MMM: Modified Monash Model
PCC: Population/concept/context
PHN: Primary Health Network
PIR: Partners in Recovery
PRISMA: Preferred Reporting Items for Systematic Reviews and Meta-Analysis extension for scoping reviews
NSW: New South Wales
NT: Northern Territory
QLD: Queensland
RHMT: Rural Health Multidisciplinary Training
SA: South Australia
TAS: Tasmania
UCC: Urgent care centre
VIC: Victoria
WA: Western Australia
