# Supplementary material for: A scoping review of the barriers and facilitators to accessing and utilising mental health services across regional, rural, and remote Australia
Source: BMC Health Serv Res. 2023 Oct 4;23:1060. doi: 10.1186/s12913-023-10034-4 (PMC10552307; doi:10.1186/s12913-023-10034-4)
Supplement: Supplementary file 1 — Additional Table 1: Search strategy for Medline Complete via EBSCO [file 12913_2023_10034_MOESM1_ESM.docx]

**Additional Table 1.**

**Search strategy for Medline Complete via EBSCO**

| **Search line** | **Seach terms** |
| --- | --- |
| #1 | (MH “mental disorder”+) |
| #2 | (TI “mental health”) |
| #3 | (TI “mental illness*”) |
| #4 | (TI “mental disorder*”) |
| #5 | (TI “mental distress”) |
| #6 | (TI “psychiatric illness*”) |
| #7 | (TI depression) |
| #8 | (TI anxiety) |
| #9 | (TI psychosis) |
| #10 | (TI “substance use”) |
| #11 | (TI “substance abuse”) |
| #12 | (TI “drug use”) |
| #13 | (TI “drug abuse”) |
| #14 | (TI “drug addiction”) |
| #15 | (TI “personality disorder*”) |
| #16 | (TI “eating disorder*”) |
| #17 | (TI schizophrenia) |
| #18 | (TI suicid*) |
| #19 | (#2 OR #3 #4 OR #5 OR #6 OR #7 OR #8 OR #9 OR #10 OR #11 OR #12 OR #13 OR #14 OR #15 OR #16 OR #17 OR #18) |
| #20 | (AB “mental health”) |
| #21 | (AB “mental illness*”) |
| #22 | (AB “mental disorder*”) |
| #23 | (AB “mental distress”) |
| #24 | (AB “psychiatric illness*”) |
| #25 | (AB depression) |
| #26 | (AB anxiety) |
| #27 | (AB psychosis) |
| #28 | (AB “substance use”) |
| #29 | (AB “substance abuse”) |
| #30 | (AB “drug use”) |
| #31 | (AB “drug abuse”) |
| #32 | (AB “drug addiction”) |
| #33 | (AB “personality disorder*”) |
| #34 | (AB “eating disorder*”) |
| #35 | (AB schizophrenia) |
| #36 | (AB suicid*) |
| #37 | (#20 OR #21 OR #22 OR #23 OR 24 #25 OR #26 OR #27 OR #28 OR #29 OR #30 OR #31 OR #32 OR #33 OR #34 OR #35 OR #36) |
| #38 | (#1 OR #19 OR #37) |
| #39 | (TI barrier*) |
| #40 | (TI obstacle*) |
| #41 | (TI challeng*) |
| #42 | (TI facilitat*) |
| #43 | (TI enabl*) |
| #44 | (TI “help seek*”) |
| #45 | (TI “help-seek*”) |
| #46 | (#39 OR #40 OR #41 OR #42 OR #43 OR #44 OR #45) |
| #47 | (AB barrier*) |
| #48 | (AB obstacl*) |
| #49 | (AB challeng*) |
| #50 | (AB facilitat*) |
| #51 | (AB enabl*) |
| #52 | (AB “help seek*”) |
| #53 | (AB “help-seek*”) |
| #54 | (#47 OR #48 OR #49 OR #50 OR #51 OR #52 OR #53) |
| #55 | (#46 OR #54) |
| #56 | (MH “rural health services”+) |
| #57 | (TI regional) |
| #58 | (TI rural) |
| #59 | (TI remote) |
| #60 | (#57 OR #58 OR #59) |
| #61 | (AB regional) |
| #62 | (AB rural) |
| #63 | (AB remote) |
| #64 | (#61 OR #62 OR #63) |
| #65 | (#56 OR #60 OR #64) |
| #66 | (MH Australia+) |
| #67 | (TI Australia) |
| #68 | (TI Victoria) |
| #69 | (TI “New South Wales”) |
| #70 | (TI NSW) |
| #71 | (TI Queensland) |
| #72 | (TI “Northern Territory”) |
| #73 | (TI NT) |
| #74 | (TI “South Australia”) |
| #75 | (TI “Western Australia) |
| #76 | (TI “Australian Capital Territory”) |
| #77 | (TI Tasmania) |
| #78 | (#67 OR #68 OR #69 OR #70 OR #71 OR #72 OR #73 OR #74 OR #75 OR #76 OR #77) |
| #79 | (AB Australia) |
| #80 | (AB Victoria) |
| #81 | (AB “New South Wales”) |
| #82 | (AB NSW) |
| #83 | (AB Queensland) |
| #84 | (AB “Northern Territory”) |
| #85 | (AB NT) |
| #86 | (AB “South Australia”) |
| #87 | (AB “Western Australia) |
| #88 | (AB “Australian Capital Territory”) |
| #89 | (AB Tasmania) |
| #90 | (#79 OR #80 OR #81 OR #82 OR #83 OR #84 OR #85 OR #86 OR #87 OR #88 OR #89) |
| #91 | (#66 OR #78 OR #90) |
| #92 | (#38 AND #55 AND #65 AND #91) |

**Search strategy for PsycINFO via EBSCO**

| **Search line** | **Seach terms** |
| --- | --- |
| #1 | (MH “mental disorders”+) |
| #2 | (TI “mental health”) |
| #3 | (TI “mental illness*”) |
| #4 | (TI “mental disorder*”) |
| #5 | (TI “mental distress”) |
| #6 | (TI “psychiatric illness*”) |
| #7 | (TI depression) |
| #8 | (TI anxiety) |
| #9 | (TI psychosis) |
| #10 | (TI “substance use”) |
| #11 | (TI “substance abuse”) |
| #12 | (TI “drug use”) |
| #13 | (TI “drug abuse”) |
| #14 | (TI “drug addiction”) |
| #15 | (TI “personality disorder*”) |
| #16 | (TI “eating disorder*”) |
| #17 | (TI schizophrenia) |
| #18 | (TI suicid*) |
| #19 | (#2 OR #3 #4 OR #5 OR #6 OR #7 OR #8 OR #9 OR #10 OR #11 OR #12 OR #13 OR #14 OR #15 OR #16 OR #17 OR #18) |
| #20 | (AB “mental health”) |
| #21 | (AB “mental illness*”) |
| #22 | (AB “mental disorder*”) |
| #23 | (AB “mental distress”) |
| #24 | (AB “psychiatric illness*”) |
| #25 | (AB depression) |
| #26 | (AB anxiety) |
| #27 | (AB psychosis) |
| #28 | (AB “substance use”) |
| #29 | (AB “substance abuse”) |
| #30 | (AB “drug use”) |
| #31 | (AB “drug abuse”) |
| #32 | (AB “drug addiction”) |
| #33 | (AB “personality disorder*”) |
| #34 | (AB “eating disorder*”) |
| #35 | (AB schizophrenia) |
| #36 | (AB suicid*) |
| #37 | (#20 OR #21 OR #22 OR #23 OR 24 #25 OR #26 OR #27 OR #28 OR #29 OR #30 OR #31 OR #32 OR #33 OR #34 OR #35 OR #36) |
| #38 | (#1 OR #19 OR #37) |
| #39 | (TI barrier*) |
| #40 | (TI obstacle*) |
| #41 | (TI challeng*) |
| #42 | (TI facilitat*) |
| #43 | (TI enabl*) |
| #44 | (TI “help seek*”) |
| #45 | (TI “help-seek*”) |
| #46 | (#39 OR #40 OR #41 OR #42 OR #43 OR #44 OR #45) |
| #47 | (AB barrier*) |
| #48 | (AB obstacl*) |
| #49 | (AB challeng*) |
| #50 | (AB facilitat*) |
| #51 | (AB enabl*) |
| #52 | (AB “help seek*”) |
| #53 | (AB “help-seek*”) |
| #54 | (#47 OR #48 OR #49 OR #50 OR #51 OR #52 OR #53) |
| #55 | (#46 OR #54) |
| #56 | (“rural health”) |
| #57 | (TI regional) |
| #58 | (TI rural) |
| #59 | (TI remote) |
| #60 | (#57 OR #58 OR #59) |
| #61 | (AB regional) |
| #62 | (AB rural) |
| #63 | (AB remote) |
| #64 | (#61 OR #62 OR #63) |
| #65 | (#56 OR #60 OR #64) |
| #66 | (Australia) |
| #67 | (TI Australia) |
| #68 | (TI Victoria) |
| #69 | (TI “New South Wales”) |
| #70 | (TI NSW) |
| #71 | (TI Queensland) |
| #72 | (TI “Northern Territory”) |
| #73 | (TI NT) |
| #74 | (TI “South Australia”) |
| #75 | (TI “Western Australia) |
| #76 | (TI “Australian Capital Territory”) |
| #77 | (TI Tasmania) |
| #78 | (#67 OR #68 OR #69 OR #70 OR #71 OR #72 OR #73 OR #74 OR #75 OR #76 OR #77) |
| #79 | (AB Australia) |
| #80 | (AB Victoria) |
| #81 | (AB “New South Wales”) |
| #82 | (AB NSW) |
| #83 | (AB Queensland) |
| #84 | (AB “Northern Territory”) |
| #85 | (AB NT) |
| #86 | (AB “South Australia”) |
| #87 | (AB “Western Australia) |
| #88 | (AB “Australian Capital Territory”) |
| #89 | (AB Tasmania) |
| #90 | (#79 OR #80 OR #81 OR #82 OR #83 OR #84 OR #85 OR #86 OR #87 OR #88 OR #89) |
| #91 | (#66 OR #78 OR #90) |
| #92 | (#38 AND #55 AND #65 AND #91) |

**Search strategy for CINAHL via EBSCO**

| **Search line** | **Seach terms** |
| --- | --- |
| #1 | (MH “mental disorders”+) |
| #2 | (TI “mental health”) |
| #3 | (TI “mental illness*”) |
| #4 | (TI “mental disorder*”) |
| #5 | (TI “mental distress”) |
| #6 | (TI “psychiatric illness*”) |
| #7 | (TI depression) |
| #8 | (TI anxiety) |
| #9 | (TI psychosis) |
| #10 | (TI “substance use”) |
| #11 | (TI “substance abuse”) |
| #12 | (TI “drug use”) |
| #13 | (TI “drug abuse”) |
| #14 | (TI “drug addiction”) |
| #15 | (TI “personality disorder*”) |
| #16 | (TI “eating disorder*”) |
| #17 | (TI schizophrenia) |
| #18 | (TI suicid*) |
| #19 | (#2 OR #3 #4 OR #5 OR #6 OR #7 OR #8 OR #9 OR #10 OR #11 OR #12 OR #13 OR #14 OR #15 OR #16 OR #17 OR #18) |
| #20 | (AB “mental health”) |
| #21 | (AB “mental illness*”) |
| #22 | (AB “mental disorder*”) |
| #23 | (AB “mental distress”) |
| #24 | (AB “psychiatric illness*”) |
| #25 | (AB depression) |
| #26 | (AB anxiety) |
| #27 | (AB psychosis) |
| #28 | (AB “substance use”) |
| #29 | (AB “substance abuse”) |
| #30 | (AB “drug use”) |
| #31 | (AB “drug abuse”) |
| #32 | (AB “drug addiction”) |
| #33 | (AB “personality disorder*”) |
| #34 | (AB “eating disorder*”) |
| #35 | (AB schizophrenia) |
| #36 | (AB suicid*) |
| #37 | (#20 OR #21 OR #22 OR #23 OR 24 #25 OR #26 OR #27 OR #28 OR #29 OR #30 OR #31 OR #32 OR #33 OR #34 OR #35 OR #36) |
| #38 | (#1 OR #19 OR #37) |
| #39 | (TI barrier*) |
| #40 | (TI obstacle*) |
| #41 | (TI challeng*) |
| #42 | (TI facilitat*) |
| #43 | (TI enabl*) |
| #44 | (TI “help seek*”) |
| #45 | (TI “help-seek*”) |
| #46 | (#39 OR #40 OR #41 OR #42 OR #43 OR #44 OR #45) |
| #47 | (AB barrier*) |
| #48 | (AB obstacl*) |
| #49 | (AB challeng*) |
| #50 | (AB facilitat*) |
| #51 | (AB enabl*) |
| #52 | (AB “help seek*”) |
| #53 | (AB “help-seek*”) |
| #54 | (#47 OR #48 OR #49 OR #50 OR #51 OR #52 OR #53) |
| #55 | (#46 OR #54) |
| #56 | (MH “rural health services”) |
| #57 | (TI regional) |
| #58 | (TI rural) |
| #59 | (TI remote) |
| #60 | (#57 OR #58 OR #59) |
| #61 | (AB regional) |
| #62 | (AB rural) |
| #63 | (AB remote) |
| #64 | (#61 OR #62 OR #63) |
| #65 | (#56 OR #60 OR #64) |
| #66 | (MH Australia+) |
| #67 | (TI Australia) |
| #68 | (TI Victoria) |
| #69 | (TI “New South Wales”) |
| #70 | (TI NSW) |
| #71 | (TI Queensland) |
| #72 | (TI “Northern Territory”) |
| #73 | (TI NT) |
| #74 | (TI “South Australia”) |
| #75 | (TI “Western Australia) |
| #76 | (TI “Australian Capital Territory”) |
| #77 | (TI Tasmania) |
| #78 | (#67 OR #68 OR #69 OR #70 OR #71 OR #72 OR #73 OR #74 OR #75 OR #76 OR #77) |
| #79 | (AB Australia) |
| #80 | (AB Victoria) |
| #81 | (AB “New South Wales”) |
| #82 | (AB NSW) |
| #83 | (AB Queensland) |
| #84 | (AB “Northern Territory”) |
| #85 | (AB NT) |
| #86 | (AB “South Australia”) |
| #87 | (AB “Western Australia) |
| #88 | (AB “Australian Capital Territory”) |
| #89 | (AB Tasmania) |
| #90 | (#79 OR #80 OR #81 OR #82 OR #83 OR #84 OR #85 OR #86 OR #87 OR #88 OR #89) |
| #91 | (#66 OR #78 OR #90) |
| #92 | (#38 AND #55 AND #65 AND #91) |

**Search strategy for Embase**

| **Search line** | **Seach terms** |
| --- | --- |
| #1 | (‘mental disease’+) |
| #2 | (TI ‘mental health’) |
| #3 | (TI ‘mental illness*’) |
| #4 | (TI ‘mental disorder*’) |
| #5 | (TI ‘mental distress’) |
| #6 | (TI ‘psychiatric illness*’) |
| #7 | (TI depression) |
| #8 | (TI anxiety) |
| #9 | (TI psychosis) |
| #10 | (TI ‘substance use’) |
| #11 | (TI ‘substance abuse’) |
| #12 | (TI ‘drug use’) |
| #13 | (TI ‘drug abuse’) |
| #14 | (TI ‘drug addiction’) |
| #15 | (TI ‘personality disorder*’) |
| #16 | (TI ‘eating disorder*’) |
| #17 | (TI schizophrenia) |
| #18 | (TI suicid*) |
| #19 | (#2 OR #3 #4 OR #5 OR #6 OR #7 OR #8 OR #9 OR #10 OR #11 OR #12 OR #13 OR #14 OR #15 OR #16 OR #17 OR #18) |
| #20 | (AB ‘mental health’) |
| #21 | (AB ‘mental illness*’) |
| #22 | (AB ‘mental disorder*’) |
| #23 | (AB ‘mental distress’) |
| #24 | (AB ‘psychiatric illness*’) |
| #25 | (AB depression) |
| #26 | (AB anxiety) |
| #27 | (AB psychosis) |
| #28 | (AB ‘substance use’) |
| #29 | (AB ‘substance abuse’) |
| #30 | (AB ‘drug use’) |
| #31 | (AB ‘drug abuse’) |
| #32 | (AB ‘drug addiction’) |
| #33 | (AB ‘personality disorder*’) |
| #34 | (AB ‘eating disorder*’) |
| #35 | (AB schizophrenia) |
| #36 | (AB suicid*) |
| #37 | (#20 OR #21 OR #22 OR #23 OR 24 #25 OR #26 OR #27 OR #28 OR #29 OR #30 OR #31 OR #32 OR #33 OR #34 OR #35 OR #36) |
| #38 | (#1 OR #19 OR #37) |
| #39 | (TI barrier*) |
| #40 | (TI obstacle*) |
| #41 | (TI challeng*) |
| #42 | (TI facilitat*) |
| #43 | (TI enabl*) |
| #44 | (TI ‘help seek*’) |
| #45 | (TI ‘help-seek*’) |
| #46 | (#39 OR #40 OR #41 OR #42 OR #43 OR #44 OR #45) |
| #47 | (AB barrier*) |
| #48 | (AB obstacl*) |
| #49 | (AB challeng*) |
| #50 | (AB facilitat*) |
| #51 | (AB enabl*) |
| #52 | (AB ‘help seek*’) |
| #53 | (AB ‘help-seek*’) |
| #54 | (#47 OR #48 OR #49 OR #50 OR #51 OR #52 OR #53) |
| #55 | (#46 OR #54) |
| #56 | (‘rural health’) |
| #57 | (TI regional) |
| #58 | (TI rural) |
| #59 | (TI remote) |
| #60 | (#57 OR #58 OR #59) |
| #61 | (AB regional) |
| #62 | (AB rural) |
| #63 | (AB remote) |
| #64 | (#61 OR #62 OR #63) |
| #65 | (#56 OR #60 OR #64) |
| #66 | (Australia+) |
| #67 | (TI Australia) |
| #68 | (TI Victoria) |
| #69 | (TI ‘New South Wales’) |
| #70 | (TI NSW) |
| #71 | (TI Queensland) |
| #72 | (TI ‘Northern Territory’) |
| #73 | (TI NT) |
| #74 | (TI ‘South Australia’) |
| #75 | (TI ‘Western Australia) |
| #76 | (TI ‘Australian Capital Territory’) |
| #77 | (TI Tasmania) |
| #78 | (#67 OR #68 OR #69 OR #70 OR #71 OR #72 OR #73 OR #74 OR #75 OR #76 OR #77) |
| #79 | (AB Australia) |
| #80 | (AB Victoria) |
| #81 | (AB ‘New South Wales’) |
| #82 | (AB NSW) |
| #83 | (AB Queensland) |
| #84 | (AB ‘Northern Territory’) |
| #85 | (AB NT) |
| #86 | (AB ‘South Australia’) |
| #87 | (AB ‘Western Australia) |
| #88 | (AB ‘Australian Capital Territory’) |
| #89 | (AB Tasmania) |
| #90 | (#79 OR #80 OR #81 OR #82 OR #83 OR #84 OR #85 OR #86 OR #87 OR #88 OR #89) |
| #91 | (#66 OR #78 OR #90) |
| #92 | (#38 AND #55 AND #65 AND #91) |

**Search strategy for SCOPUS**

| **Search line** | **Seach terms** |
| --- | --- |
| #1 | ( ABS ( ‘mental health’ ) OR ABS ( ‘mental illness*’ ) OR ABS ( ‘mental disorder*’ ) OR ABS ( ‘psychiatric illness*’ ) OR ABS ( depression ) OR ABS ( anxiety ) OR ABS ( psychosis ) OR ABS ( ‘substance use’ ) OR ABS ( ‘substance abuse’ ) OR ABS ( ‘drug use’ ) OR ABS ( ‘drug abuse’ ) OR ABS ( ‘drug addiction’ ) OR ABS ( ‘personality disorder*’ ) OR ABS ( ‘eating disorder*’ ) OR ABS ( schizophrenia ) OR ABS ( suicid* ) OR ( TITLE ( ‘mental health’ ) OR TITLE ( ‘mental illness*’ ) OR TITLE ( ‘mental disorder*’ ) OR TITLE ( ‘psychiatric illness*’ ) OR TITLE ( depression ) OR TITLE ( anxiety ) OR TITLE ( psychosis ) OR TITLE ( ‘substance use’ ) OR TITLE ( ‘substance abuse’ ) OR TITLE ( ‘drug use’ ) OR TITLE ( ‘drug abuse’ ) OR TITLE ( ‘drug addiction’ ) OR TITLE ( ‘personality disorder*’ ) OR TITLE ( ‘eating disorder*’ ) OR TITLE ( schizophrenia ) OR TITLE ( suicid* ) ) ) |
| #2 | ( ABS ( barrier* ) OR ABS ( obstacle* ) OR ABS ( challenge* ) OR ABS ( facilitat* ) OR ABS ( enabl* ) OR ABS ( ‘help seek*’ ) OR ABS ( ‘help-seek*’ ) ) ( TITLE ( barrier* ) OR TITLE ( obstacle* ) OR TITLE ( challenge* ) OR TITLE ( facilitat* ) OR TITLE ( enabl* ) OR TITLE ( ‘help seek*’ ) OR TITLE ( ‘help-seek*’ ) ) |
| #3 | ( ABS ( ‘rural health services*’ ) OR ABS ( regional ) OR ABS ( rural ) OR ABS ( remote ) ) OR ( TITLE ( ‘rural health services*’ ) OR TITLE ( regional ) OR TITLE ( rural ) OR TITLE ( remote ) ) |
| #4 | ( TITLE ( australia ) OR TITLE ( victoria ) OR TITLE ( ‘new south wales’ ) OR TITLE ( nsw ) OR TITLE ( queensland ) OR TITLE ( ‘northern territory’ ) OR TITLE ( nt ) OR TITLE ( ‘south australia’ ) OR TITLE ( ‘western australia’ ) OR TITLE ( ‘australian capital territory’ ) OR TITLE ( tasmania ) ) OR ( ABS ( australia ) OR ABS ( victoria ) OR ABS ( ‘new south wales’ ) OR ABS ( nsw ) OR ABS ( queensland ) OR ABS ( ‘northern territory’ ) OR ABS ( nt ) OR ABS ( ‘south australia’ ) OR ABS ( ‘western australia’ ) OR ABS ( ‘australian capital territory’ ) OR ABS ( tasmania ) ) |
| #5 | #1 AND #2 AND #3 AND #4 |

**Grey literature information sources**

| **Source** | **Website** |
| --- | --- |
| *Australian Commonwealth and State/Territory Websites* | |
| Australian Government Department of Health | <https://www.health.gov.au/> |
| Victoria State Government, Department of Health and Human Services | <https://www.dhhs.vic.gov.au/> |
| New South Wales State Government, Department of Health | <https://www.health.nsw.gov.au/> |
| Government of South Australia, South Australia Health | <https://www.sahealth.sa.gov.au/wps/wcm/connect/public+content/sa+health+internet/home/home> |
| Northern Territory Government, NT Health | <https://health.nt.gov.au/> |
| Government of Western Australia, Department of Health | <https://ww2.health.wa.gov.au/> |
| Tasmanian Government, Department of Health | <https://www.health.tas.gov.au/> |
| Australian Capital Territory, Health | <https://www.health.act.gov.au/> |
| *Primary Health Networks* |  |
| Central and Eastern Sydney | <https://www.cesphn.org.au/> |
| Northern Sydney | <https://sydneynorthhealthnetwork.org.au/> |
| Western Sydney | <https://wentwest.com.au/> |
| Nepean Blue Mountains | <https://www.nbmphn.com.au/> |
| South Western Sydney | <https://www.swsphn.com.au/> |
| South Eastern NSW | <https://www.coordinare.org.au/> |
| Western NSW | <https://www.wnswphn.org.au/> |
| Hunter New England and Central Coast | <https://thephn.com.au/> |
| North Coast | <https://hnc.org.au/> |
| Murrumbidgee | <https://mphn.org.au/> |
| North Western Melbourne | <https://nwmphn.org.au/> |
| Eastern Melbourne | <https://www.emphn.org.au/> |
| South Eastern Melbourne | <https://www.semphn.org.au/> |
| Gippsland | <https://www.gphn.org.au/> |
| Murray | <https://www.murrayphn.org.au/> |
| Western Victoria | <https://westvicphn.com.au/> |
| Brisbane North | <https://brisbanenorthphn.org.au/> |
| Brisbane South | <https://bsphn.org.au/> |
| Gold Coast | <https://gcphn.org.au/> |
| Darling Downs and West Moreton | <https://www.ddwmphn.com.au/> |
| Western Queensland | <https://www.wqphn.com.au/> |
| Central Queensland, Wide Bay, Sunshine Coast | <https://www.ourphn.org.au/> |
| Northern Queensland | <https://www.nqphn.com.au/> |
| Adelaide | <https://adelaidephn.com.au/> |
| Country South Australia | <https://www.countrysaphn.com.au/> |
| Western Australia | <https://www.wapha.org.au/> |
| Tasmania | <https://www.primaryhealthtas.com.au/> |
| Northern Territory | <https://www.ntphn.org.au/> |
| Australian Capital Territory | <https://www.chnact.org.au/> |
| *Rural and/or remote associations* |  |
| Services for Australian Rural and Remote Allied Health | <https://sarrah.org.au/> |
| Rural and remote health | <https://www.rrmh.com.au/> |
| National Rural Health Alliance | <https://www.ruralhealth.org.au/> |
| *Other* |  |
| Australian Institute of Health and Welfare | <https://www.aihw.gov.au/> |
| Australian Health Services Research Institute | <https://ahsri.uow.edu.au/> |
| informIT | via Deakin University |
| Australian Indigenous HealthInfoNet | <https://healthinfonet.ecu.edu.au/> |
| Suicide Prevention Australia | [https://www.suicidepreventionaust.org/#](https://www.suicidepreventionaust.org/) |
| University Departments of Rural Health | <https://arhen.org.au/> |
| Global Health Data Exchange | <http://ghdx.healthdata.org/> |
| Google | <https://www.google.com/> |
