# Supplementary material for: A scoping review of the barriers and facilitators to accessing and utilising mental health services across regional, rural, and remote Australia
Source: BMC Health Serv Res. 2023 Oct 4;23:1060. doi: 10.1186/s12913-023-10034-4 (PMC10552307; doi:10.1186/s12913-023-10034-4)
Supplement: Supplementary file 2 — Additional Table 2: Charting form used for data extraction [file 12913_2023_10034_MOESM2_ESM.docx]

**Additional Table 2. Charting form used for data extraction**

| **Descriptors** | | | | | **Population** | | | | **Concept** | | | **Context** | **Results** |
| --- | --- | --- | --- | --- | --- | --- | --- | --- | --- | --- | --- | --- | --- |
| **Author**  **& year** | **First author location** | **Study location** | **Study objective** | **Study design** | **Sample size** | **Charact-eristics (e.g., age, sex)** | **Mental health condition/**  **issue and assessment method** | **Health-**  **care provider (e.g., type/**  **role)** | **Barriers** | **Facilitators** | **Type of mental health service** | **Regional/**  **rural/**  **remote areas of Australia** | **Summary of findings** |
|  |  |  |  |  |  |  |  |  |  |  |  |  |  |
