# Supplementary material for: A scoping review of the barriers and facilitators to accessing and utilising mental health services across regional, rural, and remote Australia
Source: BMC Health Serv Res. 2023 Oct 4;23:1060. doi: 10.1186/s12913-023-10034-4 (PMC10552307; doi:10.1186/s12913-023-10034-4)
Supplement: Supplementary file 3 — Additional Table 3: Barriers and/or facilitators of access and/or utilisation factors in regional, rural, and remote Australia [file 12913_2023_10034_MOESM3_ESM.docx]

**Additional Table 3. Barriers and/or facilitators of access and/or utilisation factors in regional, rural, and remote Australia.**

| **Author citation** | **Barriers** | | **Facilitators** |
| --- | --- | --- | --- |
| ***Healthcare provider perspective*** | | | |
| Barraclough et al 2016 | *-* | | Integrated service delivery; connectedness between service enabled quicker responses; co-location facilitated systems and process integration |
| Beks et al 2018 | Limited resources; inexperienced staff and support personnel; complexity of managing multiple emergencies/presentations; vulnerability when working alone; lack of clinical skills and knowledge to assess mental health presentations; perceived insufficiency to provide patients with an appropriate level of care; limited time to build therapeutic relationships; limited inpatient options and difficulty coordinating inpatient transfers; difficulty navigating the mental health system and coordinating care for patients with acute mental health issues; insufficient after-hours telephone triage and support; difficulty coordinating care for comorbid mental health and alcohol and drug issues | | Knowing local issues and community members, acknowledging and addressing stigma; mental health education and support; closer collaborations with the community mental health team; access to an after-hours onsite mental health service; facilities and funding. |
| Clough et al 2019 | Availability of services was a significantly greater barrier for participants in outer regional/rural areas than metropolitan areas; time constraints was more of a barrier than stigma, emotional concerns, misfit of services to needs, and participation restrictions for participants in inner regional areas; time constraints was rated as a significantly greater barrier than lack of motivation, emotional concerns, negative evaluation of services, misfit of services to needs, and participation restrictions for participants in outer regional/remote areas  Access to services and professional culture were barriers across the sample, but especially among participants in regional locations; stigma was consistently reported across the sample, and was related to practitioner identity (i.e., stoicism and being able to withstand stress) among those in regional locations; recognition and awareness of stress symptoms was particularly evident among regional practitioners | | *-* |
| Cosgrave et al 2015 | Workforce shortages; staff retention; difficulty attracting staff to smaller remote towns; taking on roles beyond training level; multiple relationships; lack of 'natural' connection to the area | | *-* |
| Cosgrave et al 2018 | Difficulty with developing a profession-specific identity; challenges with providing quality multidisciplinary care; resource constraints; difficulty managing personal and professional boundaries | | *-* |
| Crotty et al, 2012 | Difficulty attracting and retaining specialist staff | | Personal nature of relationships and shared knowledge between staff in services; strong community support and connectedness; dual relationships; informal working arrangements between services |
| De Silva et al 2017 | Transport; cost; waiting times; stigma; lack of knowledge about services; geographical location; lack of rapport with healthcare provider | | Doctor-patient relationship; continuity of care; ease of access |
| Ellem et al 2019 | Insufficient resources, including a lack of youth-specific, drug and alcohol, health, and mental health services | | *-* |
| Evans et al 2020 | Resource constraints; lack of knowledge; lack of education with a willingness to engage with organisational support to overcome this issue; limited referral pathways; limited knowledge of screening tools or processes; stereotypical view of clients; perspective that comorbidity increases complexity; client expectations; professional role issues | | Willingness to engage in screening for eating disorders if provided with organisational support (i.e., education, clinical, and referral pathways, and access to screening tools) |
| Hays et al 2020 | Screening for mental health issues and suicide risk is out of scope of pharmacy role | | *-* |
| Henderson et al 2018 | Fragmentation of government responsibility; insufficient funding; bureaucracy and centralisation of service delivery | | Informal networks; sense of community; self-sufficiency |
| Hinton et al 2015 | Lack of knowledge about depression and the services available; social issues; public- and self-stigma; shame | | Specific mental health/drug and alcohol programs delivered by local health services; interservice collaboration; awareness of local resources and services; dedicated staff; a positive and open approach to service delivery |
| Isaacs et al 2017 | Lack of suitable formal supports | | *-* |
| Kidd et al 2012 | Lack of confidence; lack of accessible education | | *-* |
| Malatzky et al 2020 | Overly clinical approach; cultural assumptions of experience; family and social factors | | Non-clinical, comfortable, and informal environment; outreach options |
| Mirza et al 2019 | Difficulty with culturally appropriate assessment processes/management; cultural beliefs about medical treatment | | Use of Aboriginal Mental Health Workers, interpreters, spiritual healers, and involvement of the community elders; a culturally acceptable model of illness and utilisation of least restrictive and less coercive measures |
| Mollah et al 2018 | Differing views of the necessity of cultural competency in practice or cultural influence on mental health presentations | | Cultural competency; cultural concordance/rapport; communication; organisational attitude/culture |
| Muir-Cochrane et al 2014 | Perceived stigma; visibility within small rural communities; stoicism; perceiving others to need a service more; lack of trust; attitudes of health professionals; lack of awareness or understanding of the mental health needs of older people among health professionals; lack of service availability; service-focus on physical health; clinically-oriented mental health services; lack of knowledge about what services are available; transport; lack of collaboration between services; inefficient referral pathways; staff retention; competition for resources; gatekeeping | | *-* |
| Newman et al 2016 | Travel to the digital telehealth network; funding; low technical competency of digital telehealth networks (DTNs); privacy concerns; technical-administration issues; organisation culture and policy support; other services need to have DTN access; lack of knowledge about the system; lack of familiarity, time, and interest | | Tele-mental health reduces hospitalisations/admissions, the need for patients and staff to travel, and additional client appointments; contact with city-based psychiatrists when the rural general practitioners (GPs)/psychiatrists are unavailable; facilitation of clinical supervision; improved consultation quality and facilitation of clinical practice compared to analogue systems; increased confidence when patients do have to travel that it is necessary; immediacy of consultations; cost-savings; co-location of the digital telehealth network; improved access to local care; greater opportunity to receive care without stigma; reduced family stress; enables more anonymous care |
| Orlowski et al 2016a | Technology filters communication (e.g., limits non-verbal cues in conversation) but can be an adjunct to face-to-face practice; fast-past technological innovation hinders uptake in clinical practice; extended wait times for more rurally-living patients; limited public transport options; privacy and confidentiality concerns; reliable internet access; cost; distance to services | | Technology helps to connect to patients outside of sessions (e.g., use of SMS or email) and increases information sharing; consumer interest and willingness to engage with technology; legitimate organisational priorities, policy systems, and structures |
| Orlowski et al 2017 | Nomadic lifestyles often resulting in transferring between services in different regions; privacy; influence of a client's informal supports; diversity in organisational structures, functions, and funding; professional risk; distance to service; SMS appointment coordination and reminders can lead to inappropriate responses from clients | | Client-centred care; co-location of services; information-sharing between services; SMS appointment coordination can help with engagement and rapport |
| Procter 2015 | Consumer vulnerability (i.e., increased visibility, greater stigma, dual relationships in the community); lack of services; increased risk for clinicians and consumers when other support services (i.e., police) are lacking; technology may compensate for limited services/improve services but may not be suitable for the ageing population/unfamiliar consumers; environment is physically isolated from major services resulting in time delays to providing care or absence of services | | Person-centred and non-judgmental care that is guided by the consumer's pace, needs, and prior knowledge about the individual; appropriate and skilled communication; displaying a genuine interest in the consumer/their needs and building rapport through finding common connections/interests; normalising the consumers experience; being flexible and creative in the delivery of care; a whole-of-community approach; clinicians need to be multiskilled and adapt to the needs of the consumer when other services are absent |
| Taylor et al 2019 | Limited resources; transient workforce; funding shortages; limited service visibility | | Telehealth fills service gaps; allows expert input into care planning; patients can be managed close to home; workforce upskilling; reduces professional isolation, provides a sense of security for remote care providers; in-person contact, follow-up documentation and contact, regular case conferences, and education sessions increases visibility |
| Trail et al 2021 | Masculine norms, including stoicism; low emotional awareness; lack of skills to talk effectively about emotions | | Awareness, education, and fostering acceptable and comfortable environments between male peers |
| Wand et al 2021 | Confusion over clinical governance; challenges of a change in practice | | Physical co-location; normalisation of ED mental health presentations; improved ED care through integration communication and education; generation of patient follow-up plans; provision of therapeutic support |
| ***Service user perspective*** | |  | |
| Batterham et al 2020 | There was no difference in perceived need for help for a mental health problem between those living in metropolitan, regional, and rural locations | | *-* |
| Black et al 2012 | Perception that waiting lists are too long, especially among females but not males; perception that there are not enough mental health professionals, especially among females but not males | | *-* |
| Butterfly Foundation 2020 | No difference in the number of participants who had faced stigma within the health system as a result of an eating disorder between those in rural or metropolitan areas (F(1,424)=0.071, p=0.791); most participants experienced stigma regardless of location. Lack of resources; limited specialist services; limited training on eating disorder treatment; lack of empathy/dismissal from healthcare providers; living outside catchment zones; high turnover of healthcare workers; geographical distance to services; limited transport options; social, emotional, and financial burden of travel; costs of treatment; ineligibility for Medicare rebates for telehealth; stigma; limited awareness of mental health services; lack of specific eating disorder support; healthcare workers having pre-conceived ideas about eating disorders | | Awareness of what services are available |
| Byrne et al 2017 | Distance; lack of public transport options; lack of mental health staff and services; limited job opportunities; lack of available mental health services | | *-* |
| Dawson et al 2016 | Poor mental health literacy; guilt; delays in diagnosis; carers not being involved in care planning; reliance on service providers for information; lack of care-coordination across services; consumers and carers lack of readiness for services; lack of information for workers | | Knowledge of services; mental health literacy; familiarity in small communities; blending of professional and personal boundaries; carers being involved in care planning; knowing the mental health worker; regular, non-intrusive contact; health professionals taking responsibility for decisions that may negatively impact the consumer-carer relationship; family dynamics |
| Dunstan et al 2014 | Ethical challenges including dual-roles, confidentiality, bias and the potential for exploitation | | Personal Helpers and Mentors service (PhaMs) assists clients to access clinical services; rapport and therapeutic alliance; workers being approachable and non-judgemental; shared cultural backgrounds; workers are seen as positive examples |
| Handley 2014 | Structural factors (specifics not stated); time commitments; preference for self-management; attitudinal factors (specifics not stated); increased non-professional mental health contacts; mixed patterns of help-seeking; being female; financial disadvantage; higher education; medium or high predicted service need score; recent suicidal ideation; preparedness to use the internet for mental health treatment/information | | *-* |
| Hussain et al 2013 | Lack of timely appointments; stigma; time constraints | | *-* |
| Johnson et al 2021 | Travel; challenges of confidentiality and gossip; medical/allied health staff rotation and turnover; conservatism of the rural area | | Community; place |
| Orlowski et al 2016b | Poor internet connections; finance; knowledge of services; privacy; confidentiality; self-determination; systemic barriers; structural barriers; difficulty navigating services; services that are easy to disengage from or ill-equipped to meet mental health demands; having to repeat information due to staff/service changes; fear of the unknown; unwelcoming and non-youth friendly environments | | Personal connections; comfortable environments; workers having similar characteristics as the client (e.g., age and gender); person-centred work styles; e-mental health use as an adjunct to face-to-face interactions; prior help-seeking |
| Reynish et al 2021 | Fear of judgement; internalised stigma; fear of negativity; attitudinal bias and perceived stigma from mental health professionals; mental health professional's shortcomings; too few or no services; long waitlists; lack of waiting room anonymity; church- or faith-based services; public/systemic stigma | | Services being free of charge; self-awareness; desire to be present for others |
| Richardson et al 2015 | Sound and picture quality problems | | Therapeutic alliance; satisfaction with technical aspects of telepsychology; videoconferencing reduces proximity to therapist (due to anxious/avoidant client traits) |
| Wilson et al 2012 | Lack of mental health awareness; limited vocabulary to describe MH problems; stigma; lack of awareness about availability of services; dissatisfaction with services; staff not recognising the urgency of MH problems | |  |
| ***Combined health professional and service user perspectives*** | |  | |
| Bowman et al 2020 | Internet-based healthcare is limited, especially in relation to the quality of counselling; stigma; privacy; the mental health system is confusing to navigate; services are not specific to the needs of LGBT clients; limited internet access in rural areas; unfamiliarity with service offerings; discrimination, harassment, and rejection; proximity of community members knowing issues; few services for young people and families; concerns of physical and internet-based services being appropriately funded | | In-person support services should exist concurrently with internet-based services; promotion of services as safe spaces; internet-based services increase reach, provide privacy and anonymity, can help to combat isolation, and provide a means for early intervention |
| Consumers of Mental Health WA 2018 | Lack of service availability, especially on weekends or evenings; discomfort speaking to health workers who are known to individuals; lack of choice in services; lack of transport; distance to services; time pressures; privacy and confidentiality; stigma; lack of knowledge about services; staff retention; lack of diversity inclusion; gatekeeping; staff attitudes; financial costs; lack of culturally-suited and culturally safe services; lack of appropriate trauma and healing programs; long waitlists; limited awareness of mental health services; lack of holistic and shared-care arrangements; lack of confidence in services; gossip; under-funding and under-resourcing; high workloads; lack of referral pathways; high cost of technology and poor connectivity | | Technology as a complement to face-to-face services |
| Henderson et al 2014 | Limited service availability; lack of choice in GP services; limited access to GPs and specialist mental health services; delays in assessment diagnosis and treatment; limited transport; workforce shortages; limited capacity to meet needs of culturally and linguistically diverse (CALD) and Aboriginal communities; limited access to after-hours crisis care; telepsychiatry may not be suitable for aged care mental health clients | | Telepsychiatry; communication between services |
| Isaacs et al 2012 | Waiting time; resource constraints; lack of expertise; fear of services and service providers; difficulty in keeping appointments; lack of trust; location of environment (i.e., inside, sterile environments); lack of Indigenous staff; perceived challenges maintaining confidentiality | | Confidence in using service; selection and role of the Koori Mental Health Liaison Officer; respect and attitude; flexibility in meeting places, family centred care, service providers involvement in the community, involving Elders, familiarity with services, Indigenous staff, culturally appropriate décor, connections |
| Isaacs et al 2013 | Lack of awareness about mental health issues; men living alone; similar problems in other family members; use of traditional terms for those with signs indicating mental health issues; behaviour over-shadowing mental health problems; preference for keeping to oneself; stoicism; stereotypes; stigma; fear of being ostracised; fear of government breaking up families; lack of trust in services; racism; lack of confidentiality; fear of being labelled; lack of family support; belief that mental health problems can be overcome with alcohol and other substances; giving up; lack of purpose or role in society to women; technical language | |  |
| Mental Health Council of Tasmania 2018 | Geographical distance to services; limited range of services; service costs; transport; limited GP availability; limited mental health competence in GPs; limited psychiatry and psychology workforce in TAS; long waiting periods; reluctance to seek help; uncertainty regarding National Disability Insurance Scheme (NDIS) eligibility; high turnover of healthcare workers; NDIS roll-out leading to other service closures, casualisation of workforce, as well as loss of professional development opportunities, supervision, and career pathways; structural and cultural shifts as a result of the NDIS; lack of service funding for travel; lack of access, knowledge, or reliable technology | | Online technology; technology being used in conjunction with face-to-face services |
| Wand et al 2021 | Patients—at times—felt that their concerns were dismissed, experienced insufficient communication and long delays, were not included in decision making, and viewed staff as being unnecessarily heavy-handed; emergency department (ED) staff felt that Mental health liaison nurses (MHLNs) were reluctant to see patients <18 or those who were not considered strictly 'mental health'; some documentation incompatible with the ED | | MHLNs improve therapeutic outcomes in the ED, provide follow-up, allow privacy, provide prompt and responsive care; ED staff felt that MHLNs responded effectively to a diverse range of patient needs, added value to the NP role, were embedded within the ED team, were a specialist resource on site, provided timely and expeditious care, built ED capacity, and that the model had changed the way that the ED responded to mental health-related presentations |
| Weber et al 2012 | Having to seek help from another provider once the assessment was complete; difficulties with communication and teamwork between practitioners; isolation and lack of support; not equipped to deal with complex ED presentations; the quality of inpatient care in is limited (including by a lack of specialise training, variability of understanding and attitudes among nurses and medical staff); high workloads; lack of psychological input whilst on the medical wards; and difficulty balancing workloads within the work week | | Collaborative approach |
| ***Other*** |  | |  |
| Bridgman et al 2019 | Funding for administrative resourcing; balance of salaried vs Medicare-funded clinicians | | Reduced the need to travel to Hobart; allowed Hobart staff to be more available for young people in Hobart; reduced wait time to see a mental health clinician by 10 days |
| Duggan et al 2020 | A higher percentage of people from regional/rural areas experienced mental health access block including long waiting periods for inpatient beds, compared to those from metropolitan/urban areas | | *-* |
| Knight et al 2018; | *-* | | Collaboration; communication; engagement between service providers, carers, and clients |
| National Rural Health Alliance 2017 | Fewer psychiatrists, mental health nurses, and psychologists in regional, rural, and remote areas, compared to metropolitan areas; less Medicare Benefits Schedule expenditure for mental health services in regional, rural, and remote areas, compared to metropolitan areas | | *-* |
| Onnis et al 2020 | Lack of service capacity; incompatible data management systems; lack of procedures, processes, and systems before commencement; lack of providers of some service types and in some geographical areas; environmental issues (e.g., flood event, closure of service providers) | | Capacity to influence data management system; stakeholder feedback; ability to adapt to internal and external impacts; workforce flexibility; educating/training GPs and/or referrers in the referral process; building relationships with other service providers; innovative solutions to managing work demands |
| Salinas-Perez et al 2020 | There are nil community residential (sub-acute or long term) or day care services in the Kimberly region | | *-* |
| van Spijker et al 2019 | Low service diversity in northern WA; Western NSW had limited acute outpatient services; non-acute outpatient services were common in Country WA; acute inpatient care services were common in western NSW | | Western NSW had higher availability of non-acute outpatient services for adults; Country WA has fewer non-outpatient services; sub-acute inpatient and non-acute day services were only identified in western NSW |

*Note: NSW=New South Wales; TAS=Tasmania; WA=Western Australia; ED=emergency department; CALD=culturally and linguistically diverse; DTN=digital telehealth network; GP=general practitioner; MHLN=mental health liaison nurse; NDIS=National Disability Insurance Scheme; PHaMs=Personal Helpers and Mentors service.*
